# The role of cytokine licensing in shaping the therapeutic potential of wharton’s jelly MSCs: metabolic shift towards immunomodulation at the expense of differentiation

**DOI:** 10.1186/s13287-025-04309-2

**Published:** 2025-04-20

**Authors:** Olena Rogulska, Eliska Vavrinova, Irena Vackova, Jarmila Havelkova, Klara Gotvaldova, Pavel Abaffy, Sarka Kubinova, Michal Sima, Pavel Rossner, Lucie Bacakova, Pavla Jendelova, Katarina Smolkova, Yuriy Petrenko

**Affiliations:** 1 Department of Neuroregeneration, Institute of Experimental Medicine of the Czech Academy of Sciences, Prague, Czech Republic; 2 Laboratory of Biomaterials and Tissue Engineering, Institute of Physiology of the Czech Academy of Sciences, Prague, Czech Republic; 3 Charles University, Prague, Czech Republic; 4 Laboratory of Mitochondrial Physiology, Institute of Physiology of the Czech Academy of Sciences, Prague, Czech Republic; 5 Laboratory of Glial Biology and Omics Technologies, Institute of Biotechnology, Czech Academy of Sciences, Prague, Czech Republic; 6 Department of Optical and Biophysical Systems, Institute of Physics of the Czech Academy of Sciences, Prague, Czech Republic; 7 Department of Toxicology and Molecular Epidemiology, Institute of Experimental Medicine of the Czech Academy of Sciences, Prague, Czech Republic

**Keywords:** Multipotent mesenchymal stromal cells, Wharton’s jelly, Cytokine priming, Transcriptomics, Metabolomics, Adipogenic and osteogenic differentiation, Secretome

## Abstract

**Background:**

Cytokine licensing with pro-inflammatory molecules, such as tumour necrosis factor-alpha (TNF-α) and interferon-gamma (IFN-γ), has emerged as a promising strategy to enhance the therapeutic potential of multipotent mesenchymal stromal cells (MSCs). While licensing has demonstrated benefits for immunomodulation, its effects on other key MSC functions, including differentiation and paracrine activity, remain incompletely explored. In this study, we evaluated the transcriptomic, metabolomic, and functional changes induced by short-term TNF-α/IFN-γ priming of Wharton’s jelly-derived MSCs (WJ-MSCs).

**Methods:**

WJ-MSCs were expanded and exposed to TNF-α and IFN-γ (10 ng/ml each) for 24 h. Transcriptomic analysis was performed using RNA sequencing to identify differentially expressed genes related to immune modulation and lineage commitment. Metabolomic profiling was conducted using high-resolution mass spectrometry to assess changes in metabolic pathways. Functional assays evaluated the effects of cytokine priming on induced differentiation and growth factor secretion.

**Results:**

Cytokine licensing induced notable alterations in gene expression, upregulating pathways linked to immune response, inflammation, and cytokine signalling. However, short-term cytokine treatment significantly attenuated the osteogenic and adipogenic differentiation of MSCs, as evidenced by the reduced expression of RUNX2, ALP, CEBPA, and PPARG. The priming had a negligible effect on EGF, FGF-2, HGF, LIF, and SCF secretion. The production of VEGF-A and VEGF-C was elevated, although the levels remained low. Metabolomic analysis revealed enhanced kynurenine pathway activity, indicative of increased tryptophan catabolism, accompanied by elevated levels of fatty acids and polyamines.

**Conclusions:**

Our findings demonstrate that TNF-α/IFN-γ priming reprograms WJ-MSCs by enhancing their immunomodulatory capacity at the expense of differentiation potential. These results highlight the need for tailored strategies to optimize MSC functionality for specific clinical applications.

**Supplementary Information:**

The online version contains supplementary material available at 10.1186/s13287-025-04309-2.

## Background

Regenerative medicine is an advancing field offering substantial promise for addressing diseases that lack effective conventional treatments. Among cell-based approaches, multipotent mesenchymal stromal cell (MSC)-based has gained significant prominence. These cells, known for their ability to differentiate into osteoblasts, chondrocytes, and adipocytes, are highly valued for their potent paracrine signalling and immunomodulatory properties [1, 2]. Through the release of cytokines, growth factors, and extracellular vesicles, MSCs mediate their effect on key regenerative processes, including angiogenesis, immune modulation, and extracellular matrix remodelling [2, 3].

Wharton’s jelly-derived MSCs (WJ-MSCs), isolated from the gelatinous connective tissue of the umbilical cord, have emerged as a particularly promising cell type due to their easy accessibility through non-invasive methods. Compared to MSCs from adult tissues, WJ-MSCs exhibit a more primitive phenotype, higher proliferative potential, and enhanced plasticity, making them strong candidates for regenerative applications [1, 4]. They exhibit robust paracrine activity, secreting various trophic factors that facilitate tissue repair and immune modulation. Moreover, these cells are less affected by donor age and environmental influences, making them more consistent for clinical use. Compared to highly potent embryonic stem cells, WJ-MSCs present no ethical concerns, enhancing their appeal for therapeutic use.

Despite the mentioned advantages, WJ-MSC-based therapy faces notable challenges. Donor-to-donor variability significantly affects key therapeutic properties, including proliferation rate, differentiation capacity, and immunomodulatory activity [2, 5, 6], posing barriers to successful clinical translation.

In addition to intrinsic factors, the lack of reproducibility and high failure rates observed in advanced-stage MSC-based clinical trials are caused by extrinsic factors happening upon transplantation [2]. After injection, cells encounter hostile microenvironments characterized by inflammation, hypoxia, and altered extracellular matrix composition, which compromise cell viability, functionality, and integration. Resulting moderate clinical efficacy underscores the need for novel strategies to optimize MSC functionality and resilience.

Preconditioning strategies, such as hypoxic culture, three-dimensional culture systems, and cytokine licensing, have emerged as promising methods to enhance MSC properties by mimicking physiological or inflammatory cues encountered in vivo [3, 7, 8]. Cytokine licensing, in particular, has shown considerable promise in augmenting the therapeutic properties of MSCs. This approach involves exposing MSCs to pro-inflammatory cytokines, such as tumor necrosis factor-alpha (TNF-α) and interferon-gamma (IFN-γ), which primes the cells to synthesise higher levels of immune-regulatory molecules, e.g., indoleamine 2,3-dioxygenase (IDO), interleukin-6 (IL-6), prostaglandin E2 (PGE2), programmed death-ligand 1 (PD-L1) and human leukocyte antigen-G (HLA-G) [9, 10]. Recent studies have shown that cytokine licensing does not introduce additional donor-donor variability [11]. Moreover, the upregulation of immunomodulatory genes following cytokine licensing was remarkably synchronized despite initial donor-to-donor cell variability [12]. Harmonization of the transcriptomic profile was shown even on the single-cell level within the single-donor heterogenic MSC populations [13]. Considering the allogeneic nature of the WJ-MSC-based therapies, the achievement of synchronized cell phenotype may increase the reproducibility of treatment results.

Although cytokine licensing has demonstrated its utility in unifying immunomodulatory functions, its impact on other MSC attributes, such as differentiation capacity, metabolic activity, and secretion of growth factors, remains insufficiently characterized. Addressing these knowledge gaps is crucial to determine whether cytokine licensing can serve as a universal enhancement strategy or whether its efficacy is limited to specific applications.

In this study, we provide new insights into the effects of cytokine licensing on WJ-MSCs, mainly focusing on their adipogenic and osteogenic differentiation potential, paracrine activity, and metabolic changes. Combining transcriptomics, metabolomics, and functional assays, we aim to provide a multi-dimensional understanding of how short-term exposure to TNF-α and IFN-γ influences WJ-MSC behaviour, revealing both beneficial and detrimental effects.

## Methods

### Cell isolation, culture and licensing

Discarded human umbilical cords were obtained from healthy full-term neonates (*N* = 7) after spontaneous delivery. About 10 cm of the umbilical cord was aseptically collected, placed in sterile PBS with antibiotic–antimycotic solution at 4 °C, and transported to the laboratory within 24 h. After washing several times in PBS and brief exposure to 10% Betadine (EGIS Pharmaceuticals PLC, Budapest, Hungary), blood vessels were removed, and the remaining Wharton’s jelly tissue was chopped into small fragments (1–2 mm^3^).

WJ-MSCs were isolated from fragments (1 g) by enzymatic digestion in PBS-AA solution containing 0.26 U/ml Liberase™ TM (Roche Custom Biotech, Mannheim, Germany) and 1 mg/ml hyaluronidase at 37 °C with constant shaking for 2 hours. After removing of undigested fragments with 40-µm cell strainers, cells were centrifuged at 450 × g for 10 min. Obtained pellet was diluted in a complete culture medium containing α-MEM (Gibco^®^, Thermo Fisher Scientific), 5% pooled PL (Bioinova, Ltd.), penicillin/streptomycin (Gibco^®^, Thermo Fisher Scientific), and GlutaMAX (Gibco^®^, Thermo Fisher Scientific), and cultured at 37 °C in a humidified atmosphere, with 5% CO_2_. Regular media changes were done twice a week. After reaching near-confluence, cells were harvested using the 0.05% Trypsin/EDTA solution (Gibco^®^, Thermo Fisher Scientific) and reseeded onto a fresh plastic surface (Nunc, Roskilde, Denmark) at a density of 5 × 10^3^ cells/cm^2^. Cells at passages 3–5 were used for subsequent experiments [1, 14].

For the cytokine licensing, cells were cultured during 24 h in α-MEM, supplemented with 10% fetal bovine serum (FBS, Gibco^®^, Thermo Fisher Scientific), penicillin/streptomycin, GlutaMAX, and 10 ng/ml each of TNF-α and IFN-γ [15].

### RNA extraction and sequencing

Total RNA was extracted using the QIAGEN RNeasy Mini Kit, followed by ribosomal RNA depletion with the QIAGEN RiboMinus Eukaryote System (*N* = 7). RNA libraries were prepared with the QIAGEN QIAseq Stranded Total RNA Library Kit, which included the steps of RNA fragmentation, cDNA synthesis, end repair, adapter ligation, and PCR amplification. Sequencing was performed on an Illumina platform, generating 150 bp paired-end reads. Quality control and pre-processing involved FastQC and Trimmomatic [16, 17]. Reads were aligned to the human reference genome (GRCh38) using a STAR aligner, and transcript quantification was done with feature Counts [18]. Differential expression analysis was performed using DESeq2 [19], and genes with an adjusted *p*-value < 0.05 and log_2_ fold change > 1 were considered significant.

### Gene set enrichment analysis

**Fig. 1:**
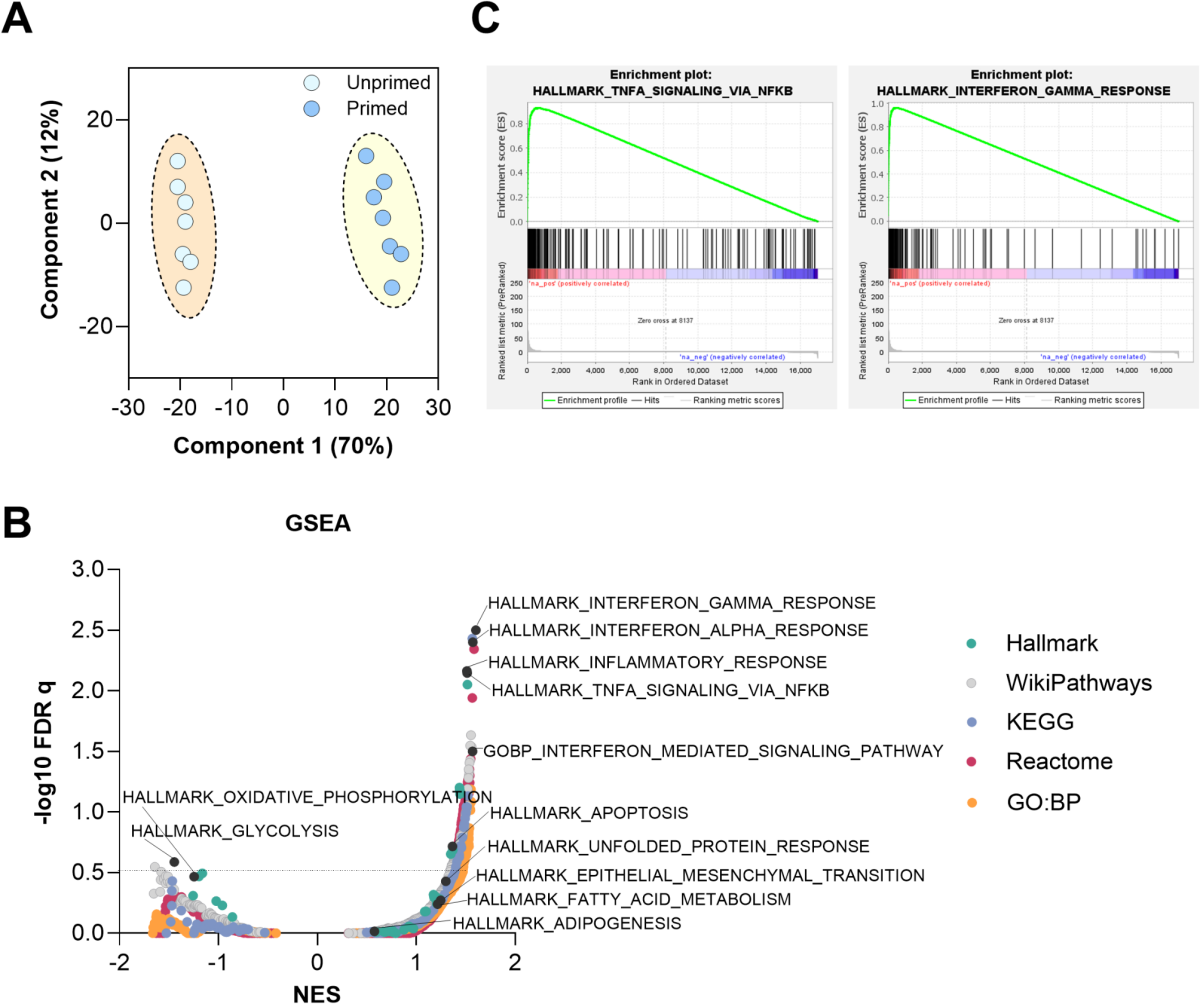
Changes in the transcriptomic profile of WJ-MSCs (*N* = 7) following the cytokine licensing: **A** – Principal component analysis (PCA) of transcriptome data from unprimed (light blue) and primed (dark blue) samples. **B** – Volcano plot depicting pathway-level enrichment analysis across the Human collections of Molecular Signatures Database (Hallmark gene sets, WikiPathways gene sets, KEGG Medicus gene sets, Reactome gene sets, and GO biological process gene sets) representing the overview of the major pathways responded to the cytokine priming. Each point represents a distinct pathway, with the x-axis showing the normalized enrichment scores (NES) and the y-axis displaying the -log10 adjusted *p*-value. The colour coding corresponds to the biological database. **C** – Enrichment plots for two hallmark gene sets significantly upregulated in primed samples: Interferon Gamma Response and TNFA signalling via NFKB pathways. Green curves represent the enrichment score (ES), while black bars indicate the position of genes within the ranked list

Data were analysed in GeneCore facility (Institute of Biotechnology of the CAS) with R programming language v4.2.2 [20]. Rlog transformed data were used in PCA and heatmap.org.Hs.eg.db v3.15.0 [21] database was used for ENSEMBL ID–gene symbol conversion. Differential gene expression was assessed using DESeq2 v1.36 package [19]. DEGs were identified using the following parameters: baseMean > 100, padj < 0.05,|log2FC| > 1. Gene set enrichment analysis (GSEA) was carried out using the GSEA software v4.3.3 [22, 23], assessed in August 2024. The gene list was ranked based on p-adjusted values. Data were compared to Human collections of Molecular Signatures Database, particularly Hallmark gene sets, WikiPathways gene sets, KEGG Medicus gene sets, Reactome gene sets, and gene ontology (GO) biological process gene sets. Calculated FDR q and NES values were used to build the graph presented in Fig. 1B. Significant terms were considered those with FDR q values < 0.25 (-log10 FDR q > 0.602) [22, 23].

### Metabolomics

The metabolomic and lipidomic profiles of cells was explored by the liquid chromatography-mass spectrometry (LC-MS) in the Metabolomics core facility at the Institute of Physiology of the CAS. For lipidomics and metabolomics, cells were grown on 6-well plates, treated as required, quickly washed with PBS, snap-frozen, and stored at -80 °C. Metabolites were extracted using a biphasic solvent system of cold methanol, methyl tert-butyl ether, and water [24].

An aliquot of the bottom (polar) phase was collected and cleaned using an acetonitrile/isopropanol mixture. After evaporation, the dry extract was resuspended in 5% methanol with 0.2% formic acid, followed by separation in an Acquity UPLC HSS T3 column (Waters). Another aliquot of the bottom phase was evaporated, resuspended in an acetonitrile/water mixture, and separated in an Acquity UPLC BEH Amide column. Metabolites were detected in negative and positive electrospray ion mode (Thermo Q Exactive Plus instrumentation) [25]. Signal intensities were normalized to the respective total ion count (TIC) before subsequent statistical analysis.

### Seahorse assay

Parallel measurement of mitochondrial respiration and glycolytic rate was performed using Seahorse XF24 Analyzer (Agilent Technologies, USA) and a modified Seahorse XF Cell Mito Stress Test. Two days before the experiment, 10^4^ cells (*N* = 6) were plated in triplicates in poly-L‐lysine‐coated assay plate wells and cultured overnight under standard culture conditions. The cell monolayers were treated with a mixture containing 10 ng/ml of TNF-α and 10 ng/ml of IFN-γ for 24 h. One hour prior to the experiment, the cells were washed with 1 ml of XF Assay Medium, i.e., 1 × DMEM lacking bicarbonate, HEPES, and glucose, and containing 2 mM glutamine, 1 mM pyruvate, and 1% FBS, pH 7.4, 37 °C. Afterward, the microplate was incubated at 37 °C. 16–24 h prior experiment, the XFe24 Sensor Cartridge was prepared by incubation with XF Calibrant at 37 °C. Basal metabolic rate without any oxidative substrate and rates after the subsequent addition of the following substrates and inhibitors were recorded: glucose (10 mM), oligomycin (Oligo, 2.5 µM), FCCP (FCCP, 4 µM), and a mixture of rotenone (Rot, 1 µM), antimycin A (AA, 0.5 µM), and 2-deoxyglucose (2DG, 100 mM). For precise normalization of rates according to cell counts, immediately after Seahorse measurements, cell nuclei were stained with Hoechst 33,342 (final concentration 1 µl / ml). Images of each well were acquired by Cytation 3 Cell Imaging Reader (BioTek) and analysed using software Gen5 (BioTek) to obtain cell counts for each well. Nuclei counts were used to normalize OCR and ECAR rates (per 10^4^ cells).

### Early osteogenic and adipogenic differentiation

The WJ-MSCs (*N* = 4, in duplicates) were seeded into 6-well plates (10^5^ cells per well), allowed to adhere overnight, and then treated by TNF-α and IFN-γ as described previously. After 24 h of cytokine treatment, the culture medium was changed to complete medium without inducers or differentiation media for adipogenic or osteogenic induction.

To induce osteogenic differentiation, the cells were cultured for 7 days in a medium composed of α-MEM supplemented with 10% FBS (Gibco^®^, Thermo Fisher Scientific), penicillin/streptomycin, GlutaMAX, 10 mM glycerol-2-phosphate, 0.1 µM dexamethasone and 100 µM L-ascorbic acid (all from Merck). The medium was changed every 2 days. After 7 days of culture, cells were collected for the qPCR analysis. In parallel, the alkaline phosphatase expression was assessed using the Fast Blue RR Salts/Naphthol kit (Merck), according to the manufacturer’s instructions, and evaluated by light microscopy.

For adipogenic differentiation, WJ-MSCs were cultured for 7 days in a medium composed of α-MEM, containing 10% FBS, penicillin/streptomycin, GlutaMAX, 0.5 mM 3-isobutyl-1-methylxanthine, 0.1 µM dexamethasone, 0.1 mM indomethacin, and 10 µg/ml insulin (all from Merck). The medium was replaced every 2 days. After 7 days of culture, cells were collected for the qPCR analysis. In parallel, the accumulation of intracellular lipid droplets was evaluated by Nile Red (1 µg/ml in PBS, Merck) staining, according to the manufacturer’s instructions. The cells were assessed using a fluorescent microscope (Leica, Germany). A quantitative analysis of the Nile Red fluorescence intensity in investigated groups was done in a microplate reader (Tecan Infinite 200; Tecan, Switzerland) at 550 nm / 640 nm wavelengths using a multiple point reading mode (100 reading points per well). The data were presented as an average Nile Red fluorescence intensity for each condition after the subtraction of negative control values (obtained from noninduced cells) and normalized by the number of cells detected by DAPI staining.

After 7 days of culture, WJ-MSCs were harvested by trypsinization and centrifuged. The pellets were washed once with PBS and stored at − 80 °C for subsequent qPCR analysis.

### Quantitative real-time PCR

To assess the efficacy of differentiation, specific human marker genes were selected:

- Osteogenic markers: RUNX2 (Runt-related transcription factor 2, Hs.PT.56a.19568141), COL1A1 (Collagen type I alpha 1 chain, Hs.PT.58.15517795), ALPL (Alkaline phosphatase, liver/bone/kidney, Hs.PT.56a.40555206) and OCN (Osteocalcin, Hs.PT.56a.39318706.g);
- Adipogenic markers: CEBPA (CCAAT/enhancer-binding protein alpha, Hs.PT.58.4022335.g) and PPARG (Peroxisome proliferator-activated receptor gamma, Hs.PT.58.25464465).

Total RNA was extracted from cell pellets using the Total RNA Purification Kit (Norgen Biotek Cor., Canada), following the manufacturer’s protocol. The concentration and purity of RNA were assessed using a NanoDrop™ 2000/2000c Spectrophotometer (Thermo Fisher Scientific). The RNase-Free DNase I Kit (Norgen Biotek Corp., Canada) was used for RNA purity improvement. RNA samples with an absorbance ratio (A260/A280) between 1.9 and 2.1 were used for subsequent experiments.

For the quantitative conversion of RNA into single-stranded cDNA, we applied a High-Capacity cDNA Reverse Transcription Kit (Thermo Fisher Scientific, USA). The reaction mix contained 1 µL 10x RT Buffer, 0.4 µL 25x dNTP Mix, 1 µL 10x RT Random Primers, 0.5 µL MultiScribe TM Reverse Transcriptase, 0.5 µL RNase Inhibitor, 1.6 µL Nuclease-free water and finally diluted RNA was added. RNA samples were normalized to the same amount of input RNA for reverse transcription. Reverse transcription was performed using the C1000 Touch™ Thermal Cycler (Bio-Rad, USA) set to these conditions: 25 °C for 10 min, 37 °C for 120 min, 85 °C for 5 min and 4 °C for the end.

The reaction mixtures were prepared using 5 µL TATTA SYBR^®^ GrandMaster^®^ Mix (TATTA Biocenter AB, Sweden), 2.6 µL RNAse free water, 0.4 µL Primers (forward and reverse primer, IDT), lastly 2 µL the cDNA was added. Quantitative real-time PCR was carried out using the CFX384 Touch Real-Time PCR Detection System (Bio-Rad, USA) following these cycling conditions: initial denaturation at 95 °C for 3 s, followed by 44 cycles of denaturation at 95 °C for 5 s, and annealing at 60 °C for 30 s extension for 72 °C for 10 s.

The following primer pairs (PrimeTime^®^ qPCR Primers, IDT) were used in the study:

**Table:** 

Genes	Forward primers (5´-3´)	Reverse primers (5´-3´)
RUNX2	CTTCACAAATCCTCCCCAAGT	AGGCGGTCAGAGAACAAAC
COL1A1	GACATGTTCAGCTTTGTGGAC	TTCTGTACGCAGGTGATTGG
ALPL	CGAGAGTGAACCATGCCA	TCCCTGATGTTATGCATGAGC
OCN	CTCACACTCCTCGCCCTAT	CGCCTGGGTCTCTTCACT
CEBPA	CCACGCCTGTCCTTAGAAAG	CCCTCCACCTTCATGTAGAAC
PPARG	GTTTCAGAAATGCCTTGCAGT	GGATTCAGCTGGTCGATATCAC
PPIA	GTGGCGGATTTGATCATTTGG	CAAGACTGAGATGCACAAGTG

All samples were in technical triplicates, and no-template controls (NTCs) were included to monitor potential contamination. Amplification efficiency and melt curve analysis were performed using CFX Maestro Software (Bio-Rad). Relative gene expression levels were calculated using the ΔΔCt method, comparing induced samples to non-induced (control) samples. PPIA (Peptidylprolyl Isomerase A, Hs.PT.58v.38887593.g) was used as the reference gene for normalization.

### Secretome composition

The growth factor concentration was assessed in the conditioned medium of primed and unprimed WJ-MSCs (*N* = 4). Briefly, MSCs at passage 3 were seeded at the density 10^5^ cells per well of a 6-well plate and cultured overnight. Then, the cytokines (TNF-α and IFN-γ at 10 ng/ml) were added to the culture medium. and the MSCs were cultured for an additional 24 h. The conditioned medium was collected and centrifuged at 1,500 rpm for 10 min and immediately stored at − 80 °C. Concentrations of EGF, FGF-2, HGF, LIF, SCF, VEGF-A, VEGF-C, IL-8, CXCL10, CCL5, MCP1, IL-6, and sVCAM-1 were assessed by Luminex^®^-based multiplex ProcartaPlex^®^ Immunoassay (Thermo Fisher Scientific Inc.), according to the manufacturer’s instructions. The measurement was performed on a Bio-Plex 200 Instrument (Bio-Rad, Prague, Czech Republic). All samples were analysed in duplicates. The cytokine and growth factor concentrations (pg/ml) were derived from the measured Mean fluorescence intensity (MFI) using fitted standard curves [14].

### Statistical analysis

#### RNA sequencing

Differential expression analysis was performed using DESeq2, and genes with an adjusted *p*-value < 0.05 and log2 fold change > 1 were considered significant.

#### Metabolomics

For volcano plots, *p*-values were calculated using Welch’s test in RStudio, version 1.4, using the matrixTests package https://CRAN.R-project.org/package=matrixTests. Multivariate analysis was performed in MetaboAnalyst 5.0 [26]. Statistically significant *p*-values are indicated in the figures as follows: *p < 0.05, **p < 0.01, ***p < 0.001, and ****p < 0.0001.

#### Seahorse analysis

After normalization according to cell count, a paired t-test was performed. The same donors were used for both the control and experimental groups. Statistically significant *p*-values are presented in figures as follows: *p < 0.05, **p < 0.01, ***p < 0.001.

#### Luminex-based immunoassay and PCR data analysis

A paired t-test was carried out to compare the levels of growth factors/cytokines or gene expression in primed and unprimed cells. The same donors were used for the control and experimental groups, ensuring a within-subjects analysis. Data analysis and visualization were performed using Prism 10.2.3. Statistically significant *p*-values are presented in figures as follows: *p < 0.05, **p < 0.01, ***p < 0.001, ****p < 0.0001.

## Results

### Effect of cytokine licensing on the transcriptomic profile of WJ-MSCs

To explore the effect of TNF-α and IFN-γ treatment on WJ-MSCs, we performed RNA sequencing and analysed differentially expressed genes.

The principal component analysis (PCA) demonstrated a robust transcriptional distinction between untreated WJ-MSCs and their cytokine-primed counterparts (Fig. 1A). Independent of the initial donor-donor variability of gene expression patterns in the untreated cells, the cytokine licensing resulted in substantial transcriptional reprogramming of WJ-MSCs and unification of their phenotype.

The cytokine licensing resulted in a significant (log2 fold change > 1 or <-1; *p* < 0.001) change in the expression of 665 genes (Additional file 1). According to the Gene Set Enrichment Analysis (GSEA) (Fig. 1B), the most upregulated pathways driven by the priming corresponded to the immune response, inflammation, and cytokine signalling indicating an enhanced immune activation in the treated cells (Fig. 1B). These findings were consistent across multiple pathway databases, including KEGG, Reactome, and GO: BP. Notably, the enrichment score (ES) for the Interferon Gamma Response and TNF-α signalling via NFKB pathways confirmed that the genes associated with these pathways were predominantly upregulated and highly ranked (Fig. 1.C). In addition, the mild activation of the apoptosis pathway was detected, indicating a normal stress response to inflammatory stimuli.

While immune-related pathways were the most prominently altered, our analysis revealed statistically non-significant trends or borderline significance changes across multiple metabolic pathways. Slight but consistent upregulation of fatty acid metabolism, unfolded protein response, shifts in expression pattern for oxidative phosphorylate, and glycolysis-related genes were detected.

Given the known interplay between metabolic reprogramming and MSC functionality, these trends warranted further investigation into their potential biological relevance.

**Fig. 2:**
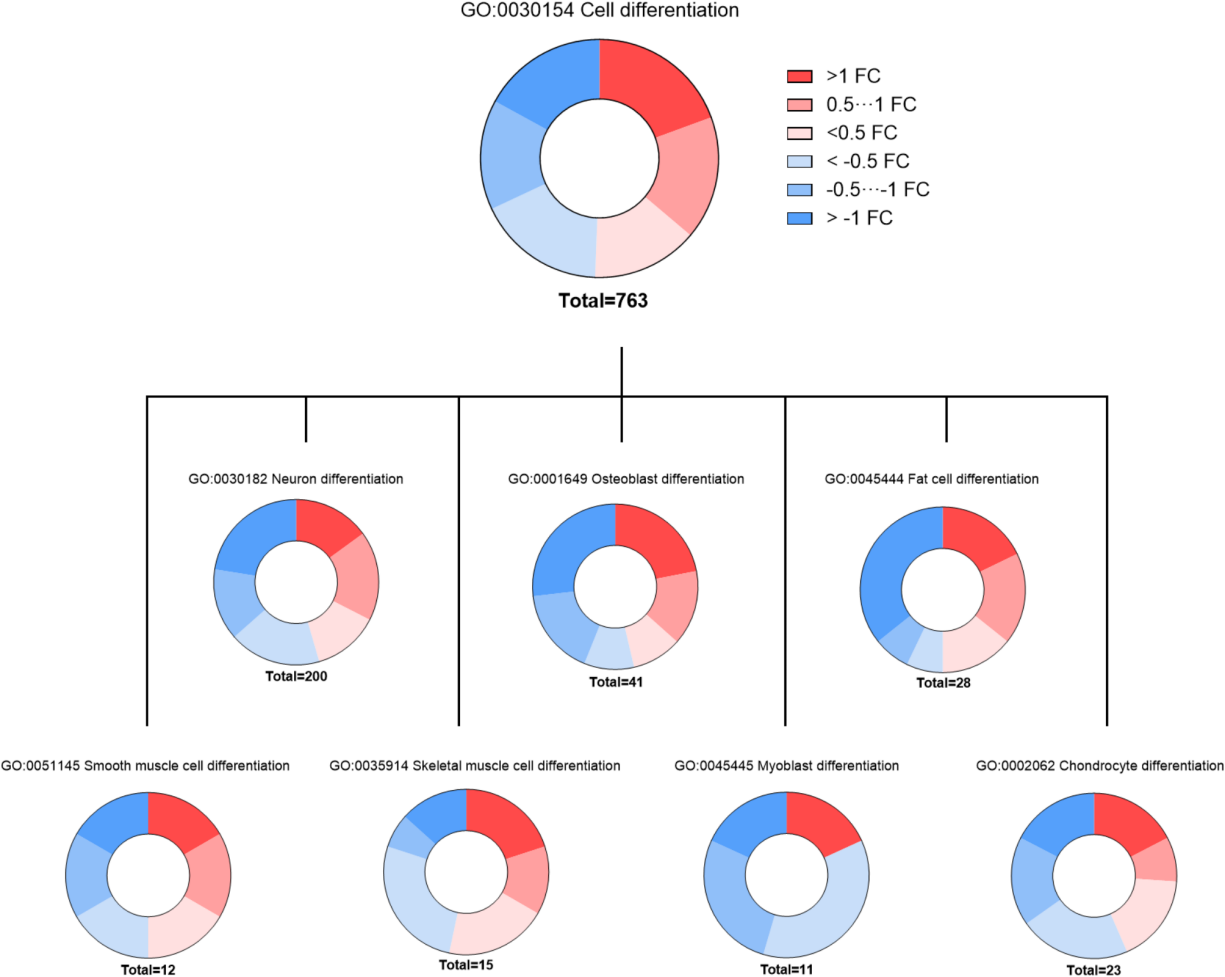
Distribution of up- and downregulated genes (log 2 fold changes, FC) referred to cell differentiation following cytokine priming based on analysis of the Gene ontology (GO) terms. The following GO terms are presented: GO:0002062 Chondrocyte differentiation, GO:0045445 Myoblast differentiation, GO:0035914 Skeletal muscle cell differentiation, GO:0051145 Smooth muscle cell differentiation, GO:0045444 Fat cell differentiation, GO:0001649 Osteoblast differentiation, GO:0030182 Neuron differentiation, GO:0030154 Cell differentiation. The identified genes were then manually categorized based on fold change ranges (> 1, 0.5–1, < 0.5, <-0.5, -0.5 to -1, and >-1), and the number of genes in each category was used for the representation

Next, we examined transcriptional changes in differentiation pathways to assess whether cytokine licensing impacts the lineage commitment of WJ-MSCs (Fig. 2) using gene ontology (GO) terms related to the theoretical differentiation potential of MSC. Within each pathway, we identified genes using a threshold of *p* < 0.05, regardless of the fold-change value. This threshold was chosen to capture a broader spectrum of genes within each selected pathway. The identified genes (Additional file 2, Table 1) were then manually categorized based on fold change ranges (> 1, 0.5–1, < 0.5, <-0.5, -0.5 to -1, and >-1), and the number of genes in each category was used for data visualization. Analysis of gene ontology (GO) terms revealed the almost equal distribution of up- and down-regulated genes within the parent cell differentiation pathway (GO:0030154). A similar tendency was observed in child GO terms, which reflects explicitly the differentiation capacity of the MSCs. Proportionate distribution of up- and downregulated genes was observed for chondrocyte (GO:0002062), osteoblast (GO:0001649), smooth muscle (GO:0051145) and neuron (GO:0030182) differentiation. In the case of the fat cell differentiation pathway (GO:0045444), despite the overall gene distribution being almost the same, more genes were allocated in the strongly downregulated cluster. The largest share of downregulated genes appeared in the myoblast differentiation pathway (GO:0045445).

Together, these results confirm the robust transcriptional reprogramming induced in MSCs by the short-term cytokine licensing. However, despite the strong activation of pathways responsible for immunomodulatory activity, no clear evidence of the positive or negative action of the priming towards the other functional properties of MSCs (i.e., differentiation-related) was detected.

### Effect of cytokine licensing on the adipogenic and osteogenic differentiation

Even subtle transcriptomic shifts in metabolic pathways or differentiation markers may reflect biologically meaningful trends that underpin the functional adaptation of MSCs to cytokine exposure.

**Fig. 3:**
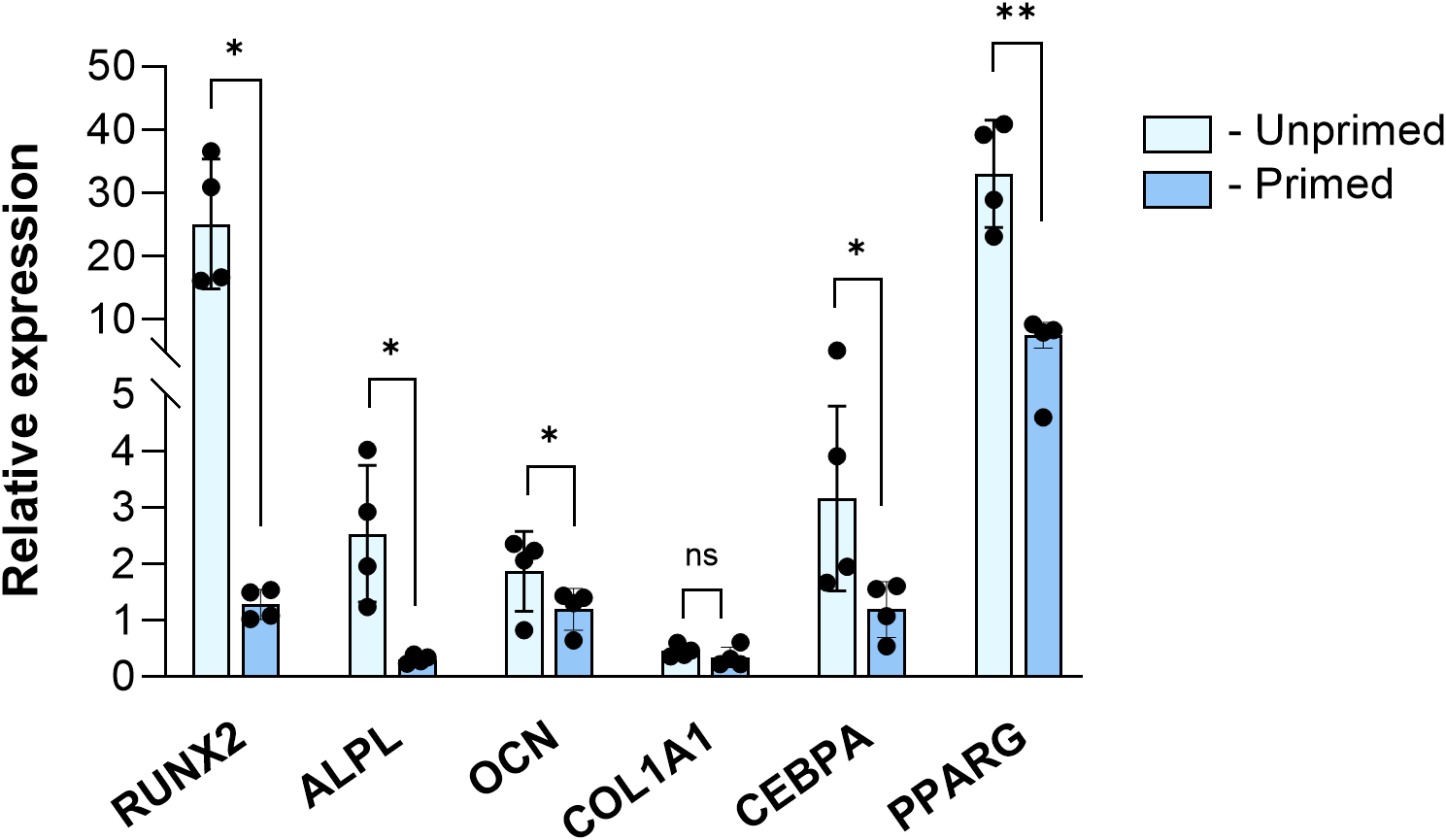
The expression of osteogenic and adipogenic genes of unprimed and cytokine licensed WJ-MSCs (*N* = 4, in duplicates) following the 7 days of the differentiation induction. Data is presented as Mean ± SD relative expression of induced cells to control cells (not subjected to differentiation induction). Note: Ns – non-significant; * - *p* < 0.05; ** - *p* < 0.01

To reveal the effect of short-term cytokine licensing on the osteogenic and adipogenic differentiation, the primed and unprimed MSCs were cultured under lineage-specific induction conditions for 7 days, and the expression of the osteogenic (*RUNX2*, *COL1A1*, *ALPL*, and *OCN*) or adipogenic (*CEBPA*, *PPARG*) genes was assessed by qPCR analysis (Fig. 3).

The significantly lower expression of early osteogenic genes (*RUNX2*,* ALPL*,* and OCN*) was detected in primed MSCs compared to their unprimed counterparts. The most remarkable differences were observed in the expression of *RUNX2* (around 20-fold), and ALPL expression was nearly abrogated. The expression of *COL1A1* was low, independent of the priming conditions (Fig. 3). No differences in the morphology between the primed and unprimed cells were detected after osteogenic induction. Only a few cells in both groups were positive for alkaline phosphatase staining (Additional file 3, Fig. 1S).

A similar cytokine licensing effect was detected during the adipogenic induction. The expression of *CEBPA* and *PPARG* genes was significantly downregulated after the short-term TNF-α and IFN-γ exposure. Similar morphological changes were detected after adipogenic induction of primed and unprimed WJ-MSCs, which were accompanied by the initiation of lipid droplet formation, confirmed by Nile red staining (Additional file 3, Fig. 1S). The quantitative analysis of Nile red fluorescence revealed a higher accumulation of lipid droplets in unprimed cultures.

These results demonstrate the inhibitory effect of the priming on the early stages of adipogenic and osteogenic differentiation of WJ-MSCs.

### Effect of cytokine licensing on the growth factor secretion

**Fig. 4:**
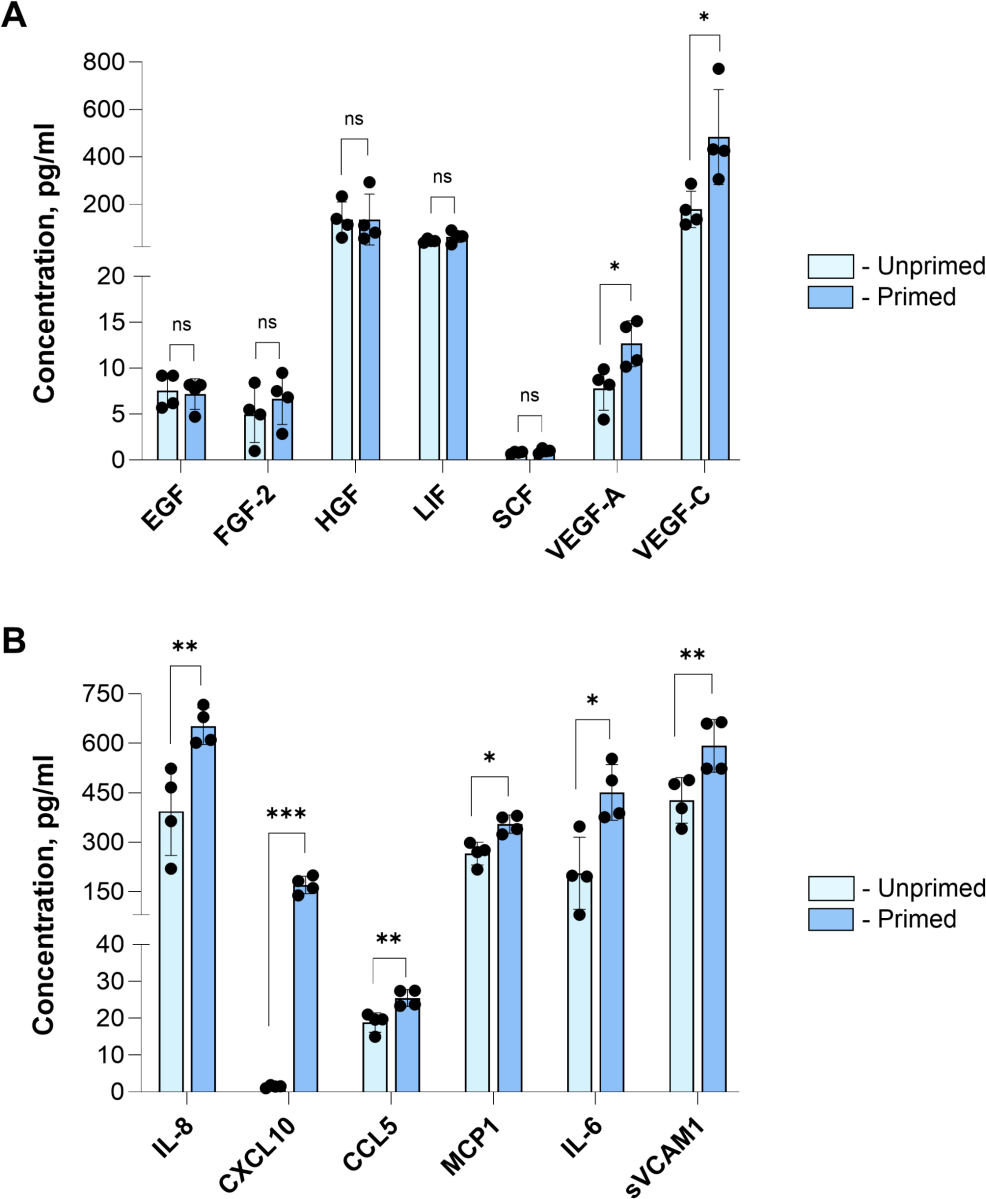
The content of secreted growth factors (**A**) and cytokines (**B**) in the conditioned medium of WJ-MSCs (*N* = 4, in duplicates). Data is presented as Mean ± SD. Note: * - *p* < 0.05

The paracrine activity of the MSCs is considered a primary driver of their therapeutic potential. To investigate potential alterations in growth factor production induced by cytokine treatment, the concentrations of six proteins (EGF, FGF-2, HGF, LIF, SCF, VEGF-A, and VEGF-C) were measured in the conditioned medium. Such growth factor panel reflects the non-immune-related therapeutic capacity of WJ-MSCs, representing their angiogenic and cytoprotective potential (Fig. 4A).

While the quantities of all growth factors were above the sensitivity threshold, the maximal content in the range of 80–100 pg/ml was identified for HGF and LIF (Fig. 4A). The cytokine licensing did not affect the production of EGF, FGF-2, HGF, LIF, and SCF. The only significant increase (*p* < 0.05) was detected for VEGF-A and VEGF-C contents in the WJ-MSCs conditioned medium following the short-term TNF-α and IFN-γ exposure.

To confirm that cytokine licensing promotes the immune-related paracrine capacity of WJ-MSCs, we assessed the content of IL-8, CXCL10, CCL5, MCP1, IL-6, and sVCAM-1 in cell-derived secretome (Fig. 4B). As a result, a significant increase in the production of all evaluated cytokines was detected, confirming the stimulatory effect of priming.

The results indicate a negligible immediate effect of cytokine priming on the production of non-immunomodulatory growth factors by WJ-MSCs.

### Effect of cytokine licensing on cell metabolism

**Fig. 5:**
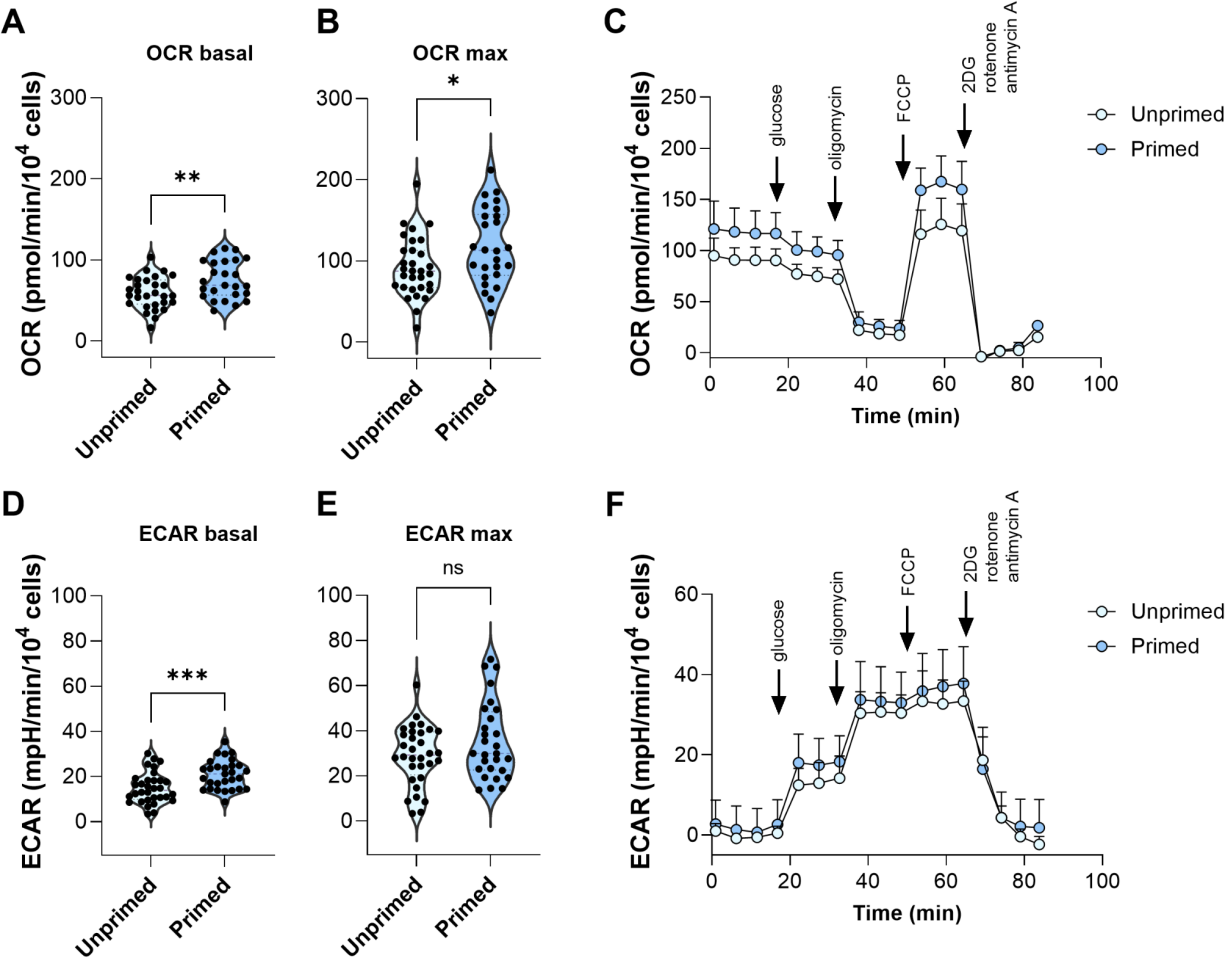
The oxygen consumption rate (OCR) (**A**, **B**) and extracellular acidification rate (ECAR) (**D**, **E**) of the unprimed and cytokine licensed WJ-MSCs (*N* = 6, in triplicates) assessed using Seahorse assay. Representative traces of OCR (**C**) and ECAR (**F**) were obtained and shown for primed and unprimed (control) cells. Note: Ns – non-significant; * - *p* < 0.05; ** - *p* < 0.01; *** - *p* < 0.001

Considering the transcriptomics data (Fig. 1B), which showed mild alteration of pathways associated with OXPHOS and glycolysis, we focused on the metabolic state of the cells. The suppressed response of MSCs to the differentiation inducers following priming and the negligible effect on growth factor production may suggest a potentially silenced energy phenotype. In this context, we analysed log2 fold change (log2 FC) of gene sets linked to energy production – glycolysis and oxidative phosphorylation. Gene expression changes for both oxidative phosphorylation and glycolysis were relatively small between the two conditions (Additional file 3, Fig. 2S). The distribution of log2 FC values revealed heterogeneity, with some genes being upregulated and some genes being downregulated. This heterogeneity suggests a possible alteration in oxygen consumption and ATP production. It is important to note that gene expression changes do not necessarily translate to functional metabolic activity. Therefore, in the next step, we analysed the metabolic activity of WJ-MSCs using the Seahorse metabolic flux analysis (Fig. 5).

The metabolic analysis of WJ-MSCs confirmed alterations following cytokine priming. Surprisingly, the oxygen consumption rate (OCR) demonstrated a significant increase in both basal and maximal levels, indicating enhanced mitochondrial activity. Concurrently, extracellular acidification rate (ECAR) analysis showed an elevation in basal levels, suggesting an upregulation in glycolytic activity. However, no significant changes were observed in maximal ECAR levels.

These findings suggest that cytokine priming may modulate the metabolic phenotype of WJ-MSCs, enhancing oxidative phosphorylation and basal glycolytic flux. Here, the TNF-α and IFN-γ treatment may induce an acute pro-inflammatory metabolic state, temporarily boosting glycolysis and mitochondrial activity to meet cellular energy demands for immune functions at the expense of other therapeutic capacities of MSCs, such as growth factor secretion and differentiation.

**Fig. 6:**
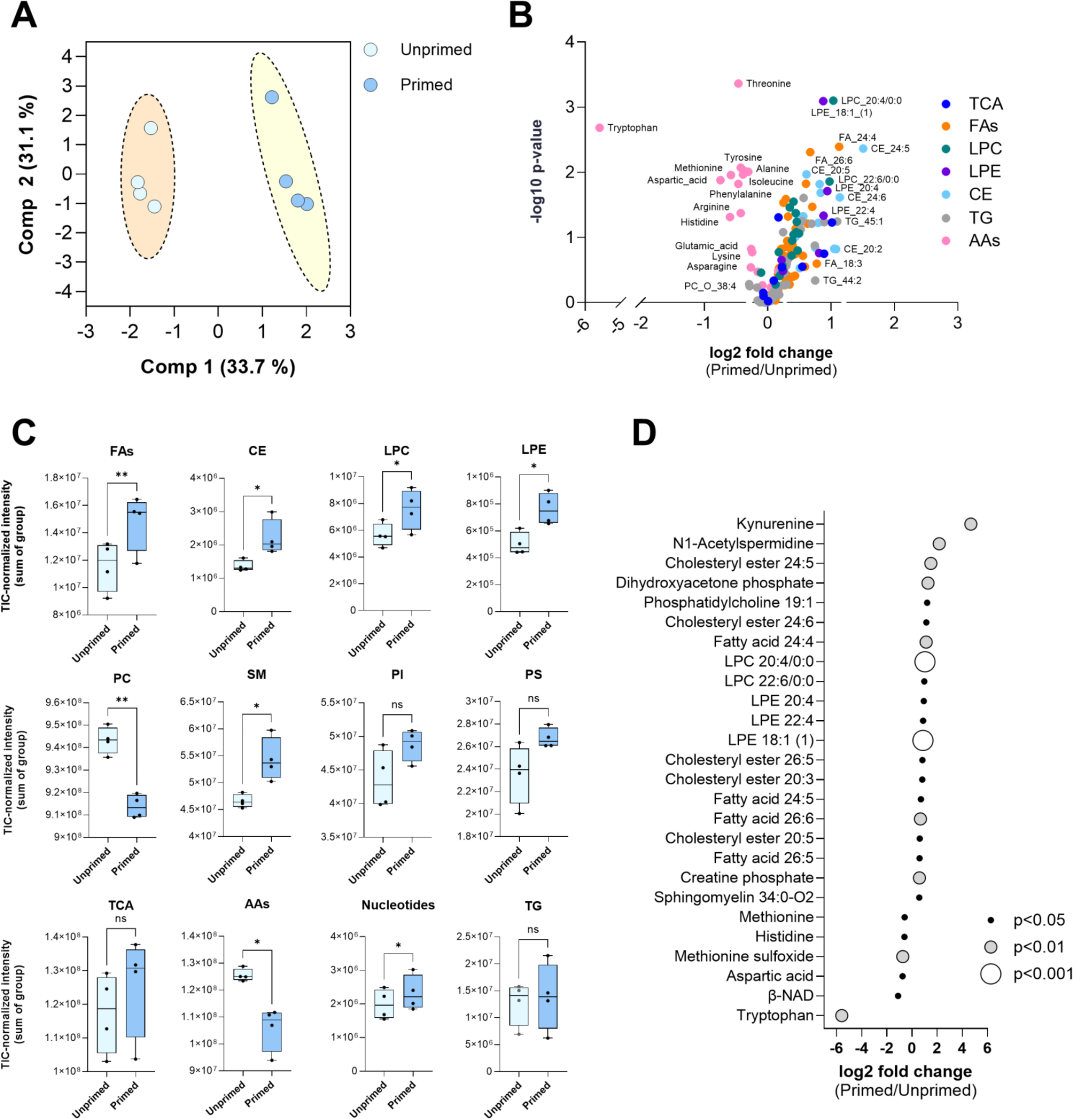
Metabolomic and lipidomic profile of WJ-MSCs following the cytokine licensing (*N* = 4, in duplicates): **A** - Principal component analysis (PCA) of metabolomic data from unprimed (light blue) and primed (dark blue) WJ-MSCs samples. **B** – Volcano plot depicting the clear separation of the main metabolite classes in WJ-MSCs induced by the cytokine priming. Each point represents a distinct most enriched metabolite within the pathway, while its colour denotes the specific metabolite class it belongs to. Abbreviations: Tricarboxylic acid cycle metabolites (TCA), triglycerides (TG), free fatty acids (FAs), lysophosphatidylcholines (LPC), lysophosphatidylethanolamines (LPE), cholesterol esters (CE), and amino acids (AAs). **C** – The changes in selected metabolite families in WJ-MSCs following the cytokine priming Note: Ns – non-significant; * - *p* < 0.05; ** - *p* < 0.01. **D** – Dot plot depicting log2 fold changes in metabolite levels between cytokine-primed WJ-MSCs and unprimed controls. Each dot represents a metabolite, with its position indicating the magnitude of fold change (log2) and its color/size denoting statistical significance: small black dot (*p* < 0.05), medium grey dot (*p* < 0.01), big white dot (*p* < 0.001)

To further assess the metabolic reprogramming of WJ-MSCs in response to cytokine licensing, we performed a global metabolomic analysis (Fig. 6). Similar to the gene expression analysis, the PCA showed a clear separation between primed and unprimed cell clusters, indicating substantial differences in metabolite profiles (Fig. 6A).

The volcano plot highlights significant changes in multiple metabolite classes in cytokine-primed versus unprimed WJ-MSCs (Fig. 6B). TNF-α and IFN-γ licensing resulted in the reduction of the free amino acid (AA) levels with simultaneous increase of the cholesterol-esters (CE) and free fatty acids (FAs). A significant decrease in the phosphatidylcholines (PCs) levels along with an increase in lysophosphatidylcholines (LPCs) and lysophosphatidylethanolamines (LPE) were observed in cells after cytokine treatment (Fig. 6C).

Finally, the dot plot (Fig. 6D) illustrates the fold changes of individual metabolites. More specifically, the most prominent changes were associated with tryptophan breakdown via the kynurenine pathway (Fig. 6D). The remarkable increase in the content of kynurenine, accompanied by the dramatic decrease in the tryptophan level, was the major event that occurred in WJ-MSCs after the cytokine exposure. In addition, the polyamine N1 -acetylspermidine level was significantly increased in primed WJ-MSCs. We observed a higher content of polyunsaturated fatty acids (PUFAs), namely FA 24:4 and FA 26:6, following the cytokine treatment (Fig. 6D). The significant upregulation of these metabolites points to enhanced amino acid catabolism and lipid turnover in primed samples.

Obtained results collectively demonstrate that inflammatory activation of WJ-MSCs by cytokine licensing is an integrated response, which co-occurs with early metabolic and differentiation changes affecting cell therapeutic potential.

## Discussion

In this study, we evaluated the effect of short-term priming of WJ-MSCs with pro-inflammatory cytokines on the cell transcriptomic and metabolomic profiles, growth factor secretion, and differentiation capacity. Consistent with the results of many research groups, TNF-α and IFN-γ induced robust change in the transcriptomic landscape of cells with a substantial shift towards anti-inflammatory immunomodulation [9–11]. Here, we decided to focus on the effect of cytokine treatment on key functional MSC characteristics, such as differentiation capacity and growth factor secretion, that are not directly involved in immunomodulation. The findings in this area remain limited, often controversial, and context-dependent.

In our study, we found that cytokine licensing significantly reshapes the transcriptomic and metabolomic profile of WJ-MSCs while suppressing their differentiation potential and having minimal impact on their paracrine regenerative capacity.

At the transcriptomic level, the impact of TNF-α and IFN-γ exposure on cell differentiation was unclear. We observed a nearly equal distribution of upregulated and downregulated genes associated with differentiation in primed cells. Heatmaps providing more detailed information on osteogenic and adipogenic gene set changes are available in the Additional materials (Additional file 3, Figs. 5S and 6S, respectively). Considering the short cytokine treatment duration (24 h), we presumed that any effects would be more noticeable during the initial stages of osteogenic and adipogenic induction, i.e. before the cells readapt to the cytokine-free culture conditions.

Notably, early osteogenic markers (*RUNX2*,* ALPL*) were significantly downregulated in cytokine-primed WJ-MSCs, while later markers (*COL1A1*,* OCN*) showed no change. Similar observations have been reported in gingival MSCs after combined exposure to TNF-α (10 ng/ml), IFN-γ (100 ng/ml), and IL-1b (1 ng/ml) [27]. Interestingly, only a few publications demonstrate the effect of dual TNF-α/IFN-γ treatment on the differentiation capacity of MSCs, while available data for individual cytokines is controversial. For instance, IFN-γ has shown both inhibitory and stimulatory effects on osteogenesis depending on the culture conditions [28, 29]. Similarly, the promotion of osteogenic differentiation by TNF-α (0.1–10 ng/ml) has been reported in [30, 31], while other studies reported that similar or higher concentrations (10–100 ng/ml) had inhibitory action [32, 33].

It is important to note that osteogenesis is usually studied after a prolonged culture of cells in the differentiation medium, containing pro-inflammatory cytokines and osteogenic inducers, thus reflecting the overall effect of chronic inflammation. However, there is limited information concerning short-term cytokine exposure used for therapeutic MSC licensing.

We can speculate that the observed suppression of osteogenesis in primed cells in our study correlated with the downregulation of the Wnt/β-catenin signalling pathway, as evidenced by reduced expression of *WNT2*,* LRP4*,* and LGR4* (Fig. 3S in the Additional file 3). The Wnt/β-catenin signalling is crucial for osteogenic differentiation of MSC [34, 35]. When activated, the β-catenin accumulates in the cytoplasm and translocates to the nuclei, regulating the target osteogenic gene expression. Wnt signalling, in turn, can be affected by the TNF-α-induced activation of NF-kB [36–38]. It was shown that the TNF-α priming induced the β-catenin degradation in human MSCs via the activation of NF-kB, leading to inhibition of the osteogenic differentiation of cells [37]. This is in line with another clinical report showing that the enhanced NF-kB signalling in systemic lupus erythematosus patients affected the Smad pathway in MSCs, which resulted in impaired osteogenesis [36]. Moreover, the blockage of NF-kB signalling pathway reversed the inhibitory effect of TNF-α, confirming the major role of this pathway in the osteogenesis [38]. In addition to TNF-α, IFN-γ by activation of JAK-STAT transcriptional factors, can interfere with β-catenin complexes, reducing their translational activity and, thus, inhibiting the Wnt signalling [39].

Indeed, the short-term exposure of WJ-MSCs to TNF-α/IFN-γ in our study led to the upregulation of *NFKB1*, *STAT1*,* JAK2*,* and JAK3* based on RNA-seq data (Fig. 3S in the Additional file 3).

In parallel, JAK-STAT activation drove significant upregulation of IDO1, which catalysed tryptophan catabolism and resulted in the accumulation of end product - kynurenine, as confirmed by our metabolomics analysis (Figs. 3S and 5D in the Additional file 3). Similarly, in the study evaluating the effect of IFN-γ on human and mouse MSCs, accumulation of kynurenine and simultaneous tryptophan depletion was observed, which led to the inhibition of osteogenesis [40].

Kynurenine has been shown to modulate downstream transcriptional activity of Aryl hydrocarbon receptor (AhR), suppressing MSC osteogenic differentiation [41]. Since IDO-mediated tryptophan catabolism represented the major change in the overall metabolomics landscape of primed WJ-MSCs, we presumed that it might be the primary mechanism contributing to impaired cell differentiation potential. To confirm this idea, we tried to rescue differentiation using 1-methyl tryptophan (1-MT), an IDO inhibitor. Surprisingly, in our study, no differences were noticed in the expression of differentiation markers compared to 1-MT-free group following 7 days of osteogenic or adipogenic induction (Fig. 4S in the Additional file 3). This lack of effect is likely due to 1-MT’s paradoxical role as an activator of AhR, independent of its IDO inhibition [42, 43]. Therefore, we may presume that the strategy of IDO blockade by synthetic inhibitors, such as 1-MT, may not be efficient enough for improving the differentiation capacity of MSCs impaired by the pro-inflammatory cytokines.

Interestingly, adipogenic differentiation was also inhibited following cytokine priming despite the downregulation of Wnt signalling and activation of AhR, both of which can promote adipogenesis [44, 45]. The opposite effect demonstrated in our study (i.e., decreased PPARγ expression) may be attributable to the dominance of other pathways activated by multifaceted effects of IFN-γ and TNF-α. It has been recently shown that cytokine licensing inhibits the adipocyte differentiation of MSCs by affecting the peroxisome proliferator-activated receptor gamma coactivator 1-alpha (PGC-1α), a master regulator of cellular antioxidant defence [46].

Indeed, our findings are consistent with the hypothesis that cytokine priming causes mild oxidative stress. As a counterweight to it, the upregulation of protective mechanisms is initiated, leading to an increase of the superoxide dismutase 2 (SOD2) gene expression (Fig. 3S in the Additional file 3). SOD2, in turn, can scavenge the mitochondrial reactive oxygen species, which promotes the adipogenic differentiation of MSCs [46, 47]. Therefore, the short-term cytokine treatment initiates the complex cascade of events, suppressing both osteogenic and adipogenic differentiation of WJ-MSCs.

Therefore, considering our data and published studies of other research groups, we may presume that the WJ-MSC licensing by the mixture of IFN-γ and TNF-α drives the complex cascade of molecular events, affecting cells’ differentiation capacity.

We have further analysed the cytokine priming’s impact on selected growth factor secretion and found minimal effects. In our study, only VEGF-A release was significantly increased but remained at low levels specific to this MSC tissue source [1]. These findings align with results shown after IFN-γ treatment of iPSC-derived MSCs during 5 days in culture [48]. In another study [49], authors demonstrated an increase in the content of VEGF-A and FGF-2 following priming of umbilical cord MSCs with TNF-α (250 U/ml, 48 h). Elevated levels of VEGF-A were detected following priming of bone marrow MSCs with TNF-α (20 ng/ml) and IFN-γ (20 ng/ml) for 24 h [50]. Our analysis showed that in contrast to VEGF-A, the FGF-2 protein level remained unchanged, yet the expression of the *FGF2* gene was significantly upregulated, suggesting post-transcriptional regulation (Fig. 3S in the Additional file 3).

It is worth noting that VEGF-C, rather than VEGF-A, has been proposed as a key factor in enhancing wound healing following IFN-γ/TNF-α priming of umbilical cord MSCs [51]. Referring to the transcriptomic analysis conducted in our study, we confirmed that the *VEGFC* gene expression was noticeably increased (Fig. 3S in the Additional file 3). Moreover, the enhanced content of the VEGF-C protein was detected in the conditioned medium of cytokine-licensed cells, compared to their unprimed counterparts (Fig. 4A).

Previously, we demonstrated that HGF production, compared to other growth factors, is dominant in WJ-MSCs [1]. In the current study, while HGF levels remained higher than VEGF-A, no significant changes were detected following TNF-α/IFN-γ exposure. Moreover, HGF gene expression was significantly downregulated (Fig. 3S in the Additional file 3), consistent with findings of the study focusing on cytokine-licensed endometrial MSCs (15 ng/ml TNF-α and 10 ng/ml IFN-γ for 48 h), where the authors reported passage-dependent *HGF* expression decrease [52]. The reduction of HGF production was detected after 72 h of priming of umbilical cord MSCs by 10 ng/mL IFN-γ and 15 ng/mL TNF-α [53].

Discrepancies in reported data concerning growth factor secretion may arise from variations in licensing methods (cytokine concentration and treatment duration) and the tissue source of MSCs.

Given the growing evidence that the secretory activity of MSCs is closely linked to their metabolic state, in the next step, we evaluated the metabolic profile of the cells by studying the mitochondrial respiration, glycolytic rate, and the changes in the metabolomics landscape. We confirmed that cytokine priming resulted in remarkable alterations of the WJ-MSCs metabolomics profile. Predictably, the most pronounced effect corresponded to IDO-mediated tryptophan breakdown via the kynurenine pathway, which is considered one of the main drivers of the immunomodulatory activity of MSCs [54].

Among the other metabolites with known effects on stem cell fate, we identified an increased level of polyamine N1-acetylspermidine in primed WJ-MSCs. This polyamine has been shown to positively influence stemness characteristics of hair follicle stromal cells [55]. Furthermore, the reduction in phosphatidylcholines (PC) content observed in our study can be associated with the decrease in the senescence levels [56]. We also found an increased content of polyunsaturated fatty acids (PUFAs) after cytokine licensing. PUFAs, beyond their notable effect on immune cells, have been shown to positively affect MSC differentiation [57]. It has also been reported that unsaturated FAs have a beneficial effect on the secretome profile of MSCs [58].

In addition, following the cytokine treatment, we observed increased cholesterol-esters (CE) levels. It is known that the cholesterol content affects the adhesion properties of MSCs and differentiation. For instance, the accumulation of the CE in mouse MSCs improved their osteogenic commitment [59, 60]. On the one hand, the increased synthesis of the PUFAs, CE, or N1-acetylspermidine may contribute to enhanced differentiation capacity of the cells or the secretion of growth factors. On the other hand, IDO-mediated tryptophan breakdown, accompanied by the remarkable accumulation of kynurenine, may lead to the inhibition of differentiation in MSCs.

While our data offer an overview of transcriptomic, metabolic, and functional changes of WJ-MSCs in response to the TNF-α / IFN-γ licensing, the presumed above molecular mechanisms necessitate additional in-depth validation at the protein level. Furthermore, considering the overall complexity of the events initiated by the cytokine treatment, uncovering the precise molecular mechanisms requires the analysis of the effect of IFN-γ and TNF-α separately with further comparison of their combinatorial action. Despite these limitations, we believe that the provided analysis may represent significant value for translational stem cell research and may be used to further improve the therapeutic performance of the MSCs.

## Conclusions

These results highlight the complex and multifaceted influence of the dual treatment of WJ-MSCs by combining IFN-γ and TNF-α. The cytokine priming simultaneously affects various signalling and metabolic pathways, which interact and compete with each other, resulting in a unique synchronized cellular phenotype. We revealed that the priming effect is dual in nature: while it enhances the immunomodulatory phenotype, it simultaneously impairs such crucial MSC function as differentiation. Here, we challenge the universality of cytokine priming as a pre-treatment strategy for diverse MSC-based therapeutic applications and emphasize the importance of further studies of complex crosstalk between pathways activated in response to priming. A comprehensive analysis of the interplay between changes in cellular processes - including gene expression, secretome release, metabolomic landscape, and bioenergetic activity - will allow finding the optimal licensing strategy required for specific clinical applications and ensure reproducible and effective outcomes.

## Electronic supplementary material

Below is the link to the electronic supplementary material.

Supplementary Material 1

Supplementary Material 2

Supplementary Material 3

## Data Availability

The data supporting this study’s findings are openly available on Zenodo under the title Data supporting the article “The role of cytokine licensing in shaping the therapeutic potential of Wharton’s jelly MSCs: a metabolic shift towards immunomodulation at the expense of differentiation.” The repository includes all figures, raw and processed transcriptomic and metabolomics data, and a list of GO terms used in this research. The dataset can be accessed at https://zenodo.org/records/14635601.

## Abbreviations

1-MT: 1-Methyl tryptophan
AA: Amino acid
AhR: Aryl hydrocarbon receptor
ALP: Alkaline phosphatase
CCL5: Chemokine ligand 5
CE: Cholesterol-esters
CEBPA: CCAAT/enhancer-binding protein alpha
COL1A1: Collagen type I alpha 1 chain
CXCL10: C-X-C motif chemokine ligand 10
ECAR: Extracellular acidification rate
EGF: Epidermal growth factor
FA: Fatty acids
FGF: Fibroblast growth factor
GSEA: Gene set enrichment analysis
GO: Gene ontology
HGF: Hepatocyte growth factor
HLA: G-human leukocyte antigen-g
IDO: Indoleamine 2,3-dioxygenase
IFN-γ: Interferon-gamma
IL: Interleukin
JAK-STAT: Janus kinase/signal transducer and activator of transcription
KEGG: Kyoto encyclopedia of genes and genomes
LIF: Leukemia inhibitory factor
LPCs: Lysophosphatidylcholines
LPEs: Lysophosphatidylethanolamines
LC-MS: Liquid chromatography-mass spectrometry
MCP-1: Monocyte chemoattractant protein-1
MSC: Multipotent mesenchymal stromal cell
NF-kB/NFKB: Nuclear factor kappa b
OCN: Osteocalcin
OCR: Oxygen consumption rate
OXPHOS: Oxidative phosphorylation
PCs: Phosphatidylcholines
PD-L1: Programmed death-ligand 1
PGE2: Prostaglandin e2
PGC-1α: Proliferator-activated receptor gamma coactivator 1-alpha
PPARG: Peroxisome proliferator-activated receptor gamma
PUFAs: Polyunsaturated fatty acids
RUNX2: Runt-related transcription factor 2
SCF: Stem cell factor
SOD2: Superoxide dismutase 2
sVCAM-1: Soluble vascular cell adhesion protein 1
TNF-α: Tumor necrosis factor-alpha
VEGF: Vascular endothelial growth factor
WJ: MSC-Wharton’s jelly multipotent mesenchymal stromal cell
