# Supplementary material for: The role of cytokine licensing in shaping the therapeutic potential of wharton’s jelly MSCs: metabolic shift towards immunomodulation at the expense of differentiation
Source: Stem Cell Res Ther. 2025 Apr 20;16:199. doi: 10.1186/s13287-025-04309-2 (PMC12010610; doi:10.1186/s13287-025-04309-2)
Supplement: Supplementary file 1 — Supplementary Material 1 [file 13287_2025_4309_MOESM1_ESM.pdf]

**Additional file 1.**

*Table 1*

The list of the most up- or down-regulated genes (log2fold >1, p<0.001), detected in RNA-seq results

| ENSEMBL | GENEID | log2FoldChange | pvalue |
|---|---|---|---|
| ENSG00000151014 | NOCT | 1.1278659623439 | 0.000941606267027992 |
| ENSG00000120833 | SOCS2 | 2.0504468752887 | 0.00092593130547183 |
| ENSG00000112276 | BVES | 1.26769850568987 | 0.000881935966655393 |
| ENSG00000174628 | IQCK | 1.06819366457118 | 0.000872913918491421 |
| ENSG00000213949 | ITGA1 | 1.2442292902673 | 0.000845922945581372 |
| ENSG00000169660 | HEXD | 1.19111853140118 | 0.000777708952185828 |
| ENSG00000120156 | TEK | 1.00645945481703 | 0.000764242274015411 |
| ENSG00000248371 | LINC02056 | 5.10761348327503 | 0.000749490407715721 |
| ENSG00000147180 | ZNF711 | 1.16002758881779 | 0.000682808020990383 |
| ENSG00000272666 | LOC105373098 | 5.75400633674126 | 0.000605936922427521 |
| ENSG00000134516 | DOCK2 | 1.37457361994248 | 0.00060453797043174 |
| ENSG00000151572 | ANO4 | 2.7909616184794 | 0.000556568060765207 |
| ENSG00000234390 | USP27X-AS1 | 5.68652655694973 | 0.000546610645622212 |
| ENSG00000197520 | FAM177B | 3.61881536144924 | 0.00052555557176319 |
| ENSG00000012211 | PRICKLE3 | 1.15101120036533 | 0.000501699202569791 |
| ENSG00000155324 | GRAMD2B | 1.07450183666277 | 0.000495487506614399 |
| ENSG00000069696 | DRD4 | 4.33131172018853 | 0.000470855317357136 |
| ENSG00000135929 | CYP27A1 | 1.93014740427748 | 0.000463256192845908 |
| ENSG00000054690 | PLEKHH1 | 1.98371931424119 | 0.000412316185557409 |
| ENSG00000083067 | TRPM3 | 4.85311860642595 | 0.000379590288876007 |
| ENSG00000141837 | CACNA1A | 1.49809899618035 | 0.000364068448288353 |
| ENSG00000164674 | SYTL3 | 1.48831686349051 | 0.00036259813345302 |
| ENSG00000073849 | ST6GAL1 | 1.54683690854983 | 0.000348003377386205 |
| ENSG00000227028 | SLC8A1-AS1 | 3.56401634012482 | 0.000306542346817078 |
| ENSG00000269720 | CCDC194 | 5.54020576121654 | 0.000305162049503625 |
| ENSG00000090376 | IRAK3 | 1.06980346573454 | 0.000284792508919551 |
| ENSG00000170745 | KCNS3 | 2.1319759716617 | 0.000283466283646417 |
| ENSG00000117594 | HSD11B1 | 3.85619421956538 | 0.000281048504041834 |
| ENSG00000250133 | HOXC-AS2 | 1.81024466883612 | 0.000253581708757258 |
| ENSG00000161921 | CXCL16 | 3.49048190574275 | 0.000222386303728324 |
| ENSG00000118946 | PCDH17 | 5.84808942136331 | 0.000199908013971467 |
| ENSG00000224577 | LINC01117 | 5.04724463484312 | 0.000198777205125794 |
| ENSG00000053747 | LAMA3 | 2.25270520587294 | 0.000192076190728554 |
| ENSG00000140379 | BCL2A1 | 2.91031562939355 | 0.000172775943172152 |
| ENSG00000168010 | ATG16L2 | 1.18859147385287 | 0.00014312617449048 |
| ENSG00000106034 | CPED1 | 1.09719949942388 | 0.000138266126744213 |
| ENSG00000168961 | LGALS9 | 1.75241146131657 | 0.000129694116261419 |
| ENSG00000137203 | TFAP2A | 2.43225794815518 | 0.000122443521309763 |
| ENSG00000241106 | HLA-DOB | 5.95615147227474 | 0.000119654036084881 |

| | | | |
|---|---|---|---|
| ENSG00000100336 | APOL4 | 4.37291096759825 | 0.000119019233308413 |
| ENSG00000065320 | NTN1 | 3.63373522454441 | 0.000118874086667775 |
| ENSG00000080561 | MID2 | 1.19314950658134 | 0.000116677218432945 |
| ENSG00000105246 | EBI3 | 4.65519490007117 | 0.000115460842292837 |
| ENSG00000183421 | RIPK4 | 5.65774408742871 | 0.000103046279713727 |
| ENSG00000137462 | TLR2 | 4.65264944086634 | 0.000101953341654442 |
| ENSG00000167995 | BEST1 | 1.30849337694627 | 9.55249378992441e-05 |
| ENSG00000234883 | MIR155HG | 2.20948996512214 | 9.45330346337193e-05 |
| ENSG00000115363 | EVA1A | 1.12706487110817 | 7.78063752723867e-05 |
| ENSG00000251136 | LOC101929709 | 1.19704217109422 | 7.38367933573985e-05 |
| ENSG00000081803 | CADPS2 | 1.95663986604037 | 7.29847719868878e-05 |
| ENSG00000277462 | ZNF670 | 1.630223099331 | 6.71408570805932e-05 |
| ENSG00000177294 | FBXO39 | 6.15231808736946 | 6.57549497357355e-05 |
| ENSG00000196371 | FUT4 | 1.08427601015209 | 6.16208881480929e-05 |
| ENSG00000070961 | ATP2B1 | 1.04272359643985 | 5.71865658791953e-05 |
| ENSG00000175592 | FOSL1 | 1.27012181494185 | 4.99156686918105e-05 |
| ENSG00000182022 | CHST15 | 2.14265857476084 | 4.93896088396958e-05 |
| ENSG00000196843 | ARID5A | 1.45391672599454 | 4.92119843533804e-05 |
| ENSG00000182957 | SPATA13 | 1.34136763437233 | 4.74736199936601e-05 |
| ENSG00000137266 | SLC22A23 | 1.59401184358631 | 4.69346807705999e-05 |
| ENSG00000172403 | SYNPO2 | 1.88375465924773 | 4.06901016432123e-05 |
| ENSG00000163554 | SPTA1 | 6.28128178800481 | 3.91663045726822e-05 |
| ENSG00000170340 | B3GNT2 | 1.09184768584566 | 3.52864898404501e-05 |
| ENSG00000003400 | CASP10 | 1.79785895983865 | 3.34668480026689e-05 |
| ENSG00000164877 | MICALL2 | 1.23012389303661 | 2.92895228209296e-05 |
| ENSG00000196358 | NTNG2 | 1.54004113425707 | 2.83553812630185e-05 |
| ENSG00000157368 | IL34 | 3.27284523559915 | 2.32298602546096e-05 |
| ENSG00000149131 | SERPING1 | 2.99816285273425 | 2.09122514181614e-05 |
| ENSG00000172817 | CYP7B1 | 5.94594085491614 | 1.93519983374156e-05 |
| ENSG00000152229 | PSTPIP2 | 1.44223246677122 | 1.87738447242821e-05 |
| ENSG00000103642 | LACTB | 1.09635226407969 | 1.84027189529928e-05 |
| ENSG00000119698 | PPP4R4 | 4.7893405807233 | 1.81052454062622e-05 |
| ENSG00000100918 | REC8 | 2.13994080108997 | 1.72112785107364e-05 |
| ENSG00000132357 | CARD6 | 1.75164294501212 | 1.56967822859369e-05 |
| ENSG00000183831 | ANKRD45 | 6.48064595114872 | 1.55679223304786e-05 |
| ENSG00000008516 | MMP25 | 6.61831673269453 | 1.52805453288794e-05 |
| ENSG00000141682 | PMAIP1 | 2.40108152931267 | 1.44651638711117e-05 |
| ENSG00000090530 | P3H2 | 1.12983889113685 | 1.29861079199562e-05 |
| ENSG00000166073 | GPR176 | 1.02640001311676 | 1.24687066820861e-05 |
| ENSG00000133687 | TMTC1 | 1.83555693253374 | 1.2402800760349e-05 |
| ENSG00000144596 | GRIP2 | 5.07969473457076 | 1.18326908549239e-05 |
| ENSG00000164308 | ERAP2 | 2.68969370017518 | 1.1711534359991e-05 |
| ENSG00000185338 | SOCS1 | 1.59397550164824 | 1.09598818915826e-05 |
| ENSG00000183153 | GJD3 | 6.09455786491865 | 1.0135895224872e-05 |
| ENSG00000150630 | VEGFC | 1.7680930959059 | 9.40487644094026e-06 |
| ENSG00000183347 | GBP6 | 6.075360528626 | 9.25868867089312e-06 |

| | | | |
|---|---|---|---|
| ENSG00000129595 | EPB41L4A | 3.24319452332398 | 8.36958506742457e-06 |
| ENSG00000146006 | LRRTM2 | 1.46116126219596 | 7.70310100556427e-06 |
| ENSG00000165949 | IFI27 | 5.11257082515827 | 7.18756755822948e-06 |
| ENSG00000108342 | CSF3 | 6.83850590308535 | 6.68128330463516e-06 |
| ENSG00000148344 | PTGES | 1.88510658783374 | 6.46019938902115e-06 |
| ENSG00000006210 | CX3CL1 | 6.67219124827696 | 5.99252516676366e-06 |
| ENSG00000137727 | ARHGAP20 | 1.33122561728959 | 5.90858521240159e-06 |
| ENSG00000147647 | DPYS | 5.66654971195784 | 5.15291164108269e-06 |
| ENSG00000166016 | ABTB2 | 1.16352814188477 | 4.85188379831507e-06 |
| ENSG00000170365 | SMAD1 | 1.0759143876213 | 4.7740405865802e-06 |
| ENSG00000204287 | HLA-DRA | 6.65806446828281 | 4.65051120065226e-06 |
| ENSG00000185404 | SP140L | 1.07002812087803 | 4.52812071305236e-06 |
| ENSG00000135373 | EHF | 7.19607634676399 | 4.15191261338528e-06 |
| ENSG00000164136 | IL15 | 1.63155562141585 | 3.70246886984201e-06 |
| ENSG00000213928 | IRF9 | 3.07438274768115 | 3.38981985955086e-06 |
| ENSG00000134198 | TSPAN2 | 3.0342609795147 | 3.26776080184334e-06 |
| ENSG00000105499 | PLA2G4C | 3.70788407651592 | 2.91394126011873e-06 |
| ENSG00000073331 | ALPK1 | 1.1865134303131 | 2.88381584330362e-06 |
| ENSG00000272269 | LOC105374952 | 3.65152643181874 | 2.45711892962567e-06 |
| ENSG00000112137 | PHACTR1 | 2.96683037821465 | 2.1531990231917e-06 |
| ENSG00000131669 | NINJ1 | 1.18901683767125 | 2.14226759332733e-06 |
| ENSG00000181804 | SLC9A9 | 1.67259301891651 | 2.10665116648107e-06 |
| ENSG00000110693 | SOX6 | 1.79072340704559 | 1.76670280195522e-06 |
| ENSG00000144810 | COL8A1 | 1.18339036290575 | 1.57923974588387e-06 |
| ENSG00000137842 | TMEM62 | 1.10744714124339 | 1.4447544626929e-06 |
| ENSG00000107968 | MAP3K8 | 1.67474676793659 | 1.3763109853634e-06 |
| ENSG00000111424 | VDR | 1.09488937514724 | 1.1982731127491e-06 |
| ENSG00000151611 | MMAA | 1.15860371762013 | 1.16819215905396e-06 |
| ENSG00000197191 | CYSRT1 | 5.77537470809513 | 1.09294691444009e-06 |
| ENSG00000254087 | LYN | 1.2500257418608 | 1.03136949066006e-06 |
| ENSG00000060491 | OGFR | 1.02069286379042 | 9.37756358720795e-07 |
| ENSG00000158859 | ADAMTS4 | 1.12749834554628 | 8.57043331350195e-07 |
| ENSG00000141052 | MYOCD | 1.54621110008814 | 8.42442441756778e-07 |
| ENSG00000173626 | TRAPPC3L | 5.57976298900865 | 8.41955202869956e-07 |
| ENSG00000101017 | CD40 | 3.35949984471495 | 5.05453978550234e-07 |
| ENSG00000143387 | CTSK | 1.72108693676258 | 4.69795586466678e-07 |
| ENSG00000118849 | RARRES1 | 1.83302164147269 | 4.66339068112077e-07 |
| ENSG00000134954 | ETS1 | 1.06658746342803 | 3.54068351056387e-07 |
| ENSG00000221926 | TRIM16 | 1.25300656126187 | 3.38102865976997e-07 |
| ENSG00000136514 | RTP4 | 4.77320370976851 | 3.36094743393453e-07 |
| ENSG00000187764 | SEMA4D | 2.42390578916788 | 3.06199439090422e-07 |
| ENSG00000153993 | SEMA3D | 1.5243007729297 | 2.32045634482412e-07 |
| ENSG00000163734 | CXCL3 | 2.53546796761968 | 1.97243052889509e-07 |
| ENSG00000168404 | MLKL | 1.11839093955446 | 1.96357139430883e-07 |
| ENSG00000146232 | NFKBIE | 2.7525921056669 | 1.70742217232938e-07 |
| ENSG00000096996 | IL12RB1 | 5.11497241119248 | 1.67261455426381e-07 |

| | | | |
|---|---|---|---|
| ENSG00000197635 | DPP4 | 1.14732612562478 | 1.62373258763165e-07 |
| ENSG00000138646 | HERC5 | 5.53408932595714 | 1.58688286956104e-07 |
| ENSG00000079335 | CDC14A | 1.34926078613695 | 1.54384942075992e-07 |
| ENSG00000106829 | TLE4 | 1.2429336769055 | 1.53041492837019e-07 |
| ENSG00000177674 | AGTRAP | 1.15854419741217 | 9.76749706361301e-08 |
| ENSG00000204261 | PSMB8-AS1 | 2.9187292301527 | 8.7033244794255e-08 |
| ENSG00000120217 | CD274 | 3.50441066658639 | 7.28256554331136e-08 |
| ENSG00000197977 | ELOVL2 | 2.15278429984613 | 6.9357693251954e-08 |
| ENSG00000143106 | PSMA5 | 1.0144309567635 | 6.36117032255105e-08 |
| ENSG00000198832 | SELENOM | 1.20548982698604 | 4.79915137353293e-08 |
| ENSG00000206337 | HCP5 | 5.0054616014583 | 4.6139418707416e-08 |
| ENSG00000125430 | HS3ST3B1 | 2.22259322607863 | 4.51339200763381e-08 |
| ENSG00000110492 | MDK | 1.0205373786236 | 4.30547858992214e-08 |
| ENSG00000028277 | POU2F2 | 1.20386759678885 | 3.88682693753501e-08 |
| ENSG00000099998 | GGT5 | 1.6241020996559 | 3.72670574607758e-08 |
| ENSG00000185507 | IRF7 | 2.52337725077893 | 3.09658025111597e-08 |
| ENSG00000136490 | LIMD2 | 1.84135658948879 | 2.51856381295555e-08 |
| ENSG00000130066 | SAT1 | 1.41829266603941 | 2.23734515832838e-08 |
| ENSG00000102554 | KLF5 | 1.89572730638731 | 2.17060183367843e-08 |
| ENSG00000181617 | FDCSP | 8.93108257176734 | 2.02521854299574e-08 |
| ENSG00000168016 | TRANK1 | 1.57741127203515 | 1.94465183089933e-08 |
| ENSG00000108771 | DHX58 | 2.74957085395859 | 1.48757218626855e-08 |
| ENSG00000128203 | ASPHD2 | 2.03722153349538 | 1.4826371426773e-08 |
| ENSG00000178860 | MSC | 3.14244301969032 | 1.40607320788539e-08 |
| ENSG00000204397 | CARD16 | 1.67992869980284 | 1.30529203920258e-08 |
| ENSG00000243811 | APOBEC3D | 3.02088495184325 | 1.27487620773326e-08 |
| ENSG00000163347 | CLDN1 | 6.23551366898877 | 1.18133880918729e-08 |
| ENSG00000111266 | DUSP16 | 1.3342383497992 | 1.05242783449059e-08 |
| ENSG00000120526 | NUDCD1 | 1.00755651450974 | 9.89791414799214e-09 |
| ENSG00000158773 | USF1 | 1.00573198096931 | 7.56936951622328e-09 |
| ENSG00000154721 | JAM2 | 2.25422683762138 | 6.80366374836027e-09 |
| ENSG00000050730 | TNIP3 | 2.8124374885313 | 6.78377765307773e-09 |
| ENSG00000124549 | BTN2A3P | 1.37691896018362 | 6.46072578673332e-09 |
| ENSG00000110330 | BIRC2 | 1.01615276712489 | 5.08928299476638e-09 |
| ENSG00000127191 | TRAF2 | 1.41941150093064 | 4.96517377373328e-09 |
| ENSG00000204516 | MICB | 1.7879903128343 | 2.43303895836806e-09 |
| ENSG00000158286 | RNF207 | 1.94779864865561 | 1.9452599188378e-09 |
| ENSG00000133805 | AMPD3 | 1.30985979130581 | 1.93143382700073e-09 |
| ENSG00000116194 | ANGPTL1 | 1.93858318596754 | 1.85072427389887e-09 |
| ENSG00000171729 | TMEM51 | 1.46644347322614 | 1.74743963367901e-09 |
| ENSG00000066583 | ISOC1 | 1.02407872997122 | 1.4607558555401e-09 |
| ENSG00000133321 | PLAAT4 | 5.71749441476515 | 1.36563297725199e-09 |
| ENSG00000205885 | C1RL-AS1 | 1.34482919307702 | 1.17182276135021e-09 |
| ENSG00000132003 | ZSWIM4 | 1.37871250931537 | 1.07921122409636e-09 |
| ENSG00000159128 | IFNGR2 | 1.01731285443956 | 1.06709930506368e-09 |
| ENSG00000144837 | PLA1A | 7.26617198990162 | 1.02015020201019e-09 |

| | | | |
|---|---|---|---|
| ENSG00000160932 | LY6E | 1.08400124521356 | 9.79693116167539e-10 |
| ENSG00000105835 | NAMPT | 1.22025376438586 | 6.29229101699105e-10 |
| ENSG00000136052 | SLC41A2 | 1.33088371526975 | 5.07768740423392e-10 |
| ENSG00000172575 | RASGRP1 | 6.60385931664907 | 4.69034441211705e-10 |
| ENSG00000028137 | TNFRSF1B | 2.35910485425244 | 4.2838686936928e-10 |
| ENSG00000166801 | FAM111A | 1.01675625353296 | 3.9644074898318e-10 |
| ENSG00000134321 | RSAD2 | 6.11822718391184 | 3.85812488516928e-10 |
| ENSG00000104312 | RIPK2 | 1.782336870373 | 3.16340071511561e-10 |
| ENSG00000125730 | C3 | 6.56607126153943 | 2.94518189268112e-10 |
| ENSG00000169621 | APLF | 1.40504285164764 | 2.84073201591277e-10 |
| ENSG00000142961 | MOB3C | 1.44922701925155 | 2.70309073395658e-10 |
| ENSG00000206190 | ATP10A | 1.77668896662561 | 2.56847483182715e-10 |
| ENSG00000166920 | C15orf48 | 6.93549275893957 | 2.50060063835134e-10 |
| ENSG00000125826 | RBCK1 | 1.02674983771313 | 1.98295148365357e-10 |
| ENSG00000137571 | SLCO5A1 | 4.54483925810326 | 1.57942738404537e-10 |
| ENSG00000205413 | SAMD9 | 1.90329653498227 | 1.53263270011893e-10 |
| ENSG00000135905 | DOCK10 | 1.19967111782259 | 1.49372869202019e-10 |
| ENSG00000152689 | RASGRP3 | 6.30903744025233 | 1.46837182812064e-10 |
| ENSG00000105639 | JAK3 | 2.40365148737099 | 1.29133729601067e-10 |
| ENSG00000164430 | CGAS | 1.39470412061381 | 1.26095607162617e-10 |
| ENSG00000138685 | FGF2 | 1.36007699319376 | 1.19848461031997e-10 |
| ENSG00000123992 | DNPEP | 1.41583421663187 | 1.1907864439607e-10 |
| ENSG00000120337 | TNFSF18 | 7.03987862046234 | 1.12225258964721e-10 |
| ENSG00000168899 | VAMP5 | 1.65553748029281 | 1.04038048887784e-10 |
| ENSG00000126561 | STAT5A | 1.65663780003344 | 1.02970147437136e-10 |
| ENSG00000114423 | CBLB | 1.18743131507798 | 1.00337702200949e-10 |
| ENSG00000197646 | PDCD1LG2 | 2.07620507117494 | 7.98871095195003e-11 |
| ENSG00000198604 | BAZ1A | 1.02754989641957 | 5.20512871555755e-11 |
| ENSG00000183486 | MX2 | 3.13467430794919 | 5.15846468980879e-11 |
| ENSG00000058866 | DGKG | 5.64762648124016 | 3.91137852007211e-11 |
| ENSG00000173114 | LRRN3 | 4.14968996404837 | 3.6317234977939e-11 |
| ENSG00000171608 | PIK3CD | 1.37842712554253 | 3.36118633829508e-11 |
| ENSG00000134326 | CMPK2 | 5.75015356758205 | 2.69722698486412e-11 |
| ENSG00000146859 | TMEM140 | 4.81410685604932 | 2.54370036983785e-11 |
| ENSG00000178882 | RFLNA | 2.4565072972565 | 2.33939786106637e-11 |
| ENSG00000105339 | DENND3 | 1.45343210913477 | 1.95914702058689e-11 |
| ENSG00000121858 | TNFSF10 | 7.3271914285067 | 1.60968627569017e-11 |
| ENSG00000135114 | OASL | 5.28826082397122 | 1.36405569707464e-11 |
| ENSG00000130487 | KLHDC7B | 6.03109319240224 | 1.31085060091526e-11 |
| ENSG00000137124 | ALDH1B1 | 1.25552944494615 | 1.19037120627347e-11 |
| ENSG00000136244 | IL6 | 4.61689708836973 | 1.15160816040538e-11 |
| ENSG00000136205 | TNS3 | 1.36179259444956 | 1.11949966875886e-11 |
| ENSG00000172183 | ISG20 | 3.90014177032716 | 8.70844167709546e-12 |
| ENSG00000165895 | ARHGAP42 | 1.37414979848543 | 8.1354866228441e-12 |
| ENSG00000081041 | CXCL2 | 2.73144430961171 | 5.80757080886226e-12 |
| ENSG00000029153 | ARNTL2 | 1.28726613192655 | 5.1665975870197e-12 |

| | | | |
|---|---|---|---|
| ENSG00000128849 | CGNL1 | 2.11721332014202 | 4.74064564157173e-12 |
| ENSG00000128284 | APOL3 | 4.15329860268324 | 4.41197468946912e-12 |
| ENSG00000166881 | NEMP1 | 1.2162476054723 | 3.69859515179937e-12 |
| ENSG00000166592 | RRAD | 5.62508713263 | 2.83524701866438e-12 |
| ENSG00000049249 | TNFRSF9 | 5.33857062373063 | 2.60010492204827e-12 |
| ENSG00000120498 | TEX11 | 7.74664746498842 | 1.78405263514864e-12 |
| ENSG00000117069 | ST6GALNAC5 | 1.59553185750491 | 1.48370727110906e-12 |
| ENSG00000065060 | UHRF1BP1 | 1.33193362966923 | 1.0319553413438e-12 |
| ENSG00000179583 | CIITA | 6.78245007932864 | 9.22537431287347e-13 |
| ENSG00000122877 | EGR2 | 3.19159329173212 | 8.37304609387647e-13 |
| ENSG00000139192 | TAPBPL | 2.60935668435578 | 8.25554353079251e-13 |
| ENSG00000048052 | HDAC9 | 2.95676840448643 | 7.21115963147975e-13 |
| ENSG00000172123 | SLFN12 | 1.19871068774138 | 5.19661027963124e-13 |
| ENSG00000163755 | HPS3 | 1.04856293151449 | 4.24674293825216e-13 |
| ENSG00000154655 | L3MBTL4 | 7.06155880656404 | 3.47156484805914e-13 |
| ENSG00000100985 | MMP9 | 8.74553542752449 | 2.98226941636726e-13 |
| ENSG00000132481 | TRIM47 | 2.99660862375441 | 2.76911554666615e-13 |
| ENSG00000165806 | CASP7 | 1.23459702364813 | 2.41171948556613e-13 |
| ENSG00000167191 | GPRC5B | 3.38487699144013 | 2.39658351192062e-13 |
| ENSG00000110218 | PANX1 | 1.13016544358406 | 2.00513572435935e-13 |
| ENSG00000168062 | BATF2 | 5.44376901921291 | 1.80616110886107e-13 |
| ENSG00000138448 | ITGAV | 1.09970190273615 | 1.1687988979183e-13 |
| ENSG00000138435 | CHRNA1 | 5.99006997287951 | 9.73553446385992e-14 |
| ENSG00000088387 | DOCK9 | 1.36332573786816 | 8.82747394392679e-14 |
| ENSG00000164619 | BMPER | 1.37438775963337 | 5.70686477701797e-14 |
| ENSG00000155465 | SLC7A7 | 2.70386733335155 | 4.52155348260249e-14 |
| ENSG00000119714 | GPR68 | 3.17588340158559 | 3.20829419700247e-14 |
| ENSG00000197721 | CR1L | 7.75608991362372 | 3.06960577801282e-14 |
| ENSG00000167034 | NKX3-1 | 3.32782041930788 | 2.93784181477859e-14 |
| ENSG00000137767 | SQOR | 1.85603901389398 | 1.73637438250042e-14 |
| ENSG00000180628 | PCGF5 | 1.42644406788667 | 1.60616801337235e-14 |
| ENSG00000168386 | FILIP1L | 1.88668906494412 | 1.00298768395348e-14 |
| ENSG00000115008 | IL1A | 2.46787282008439 | 9.96563065296521e-15 |
| ENSG00000085449 | WDFY1 | 1.01797453254496 | 7.49594032805924e-15 |
| ENSG00000145147 | SLIT2 | 1.1417857260431 | 5.93283002898002e-15 |
| ENSG00000136732 | GYPC | 1.20553046826866 | 3.85112154540324e-15 |
| ENSG00000178685 | PARP10 | 3.24319884277265 | 3.57191008075201e-15 |
| ENSG00000185245 | GP1BA | 7.24219386170273 | 3.56043889024305e-15 |
| ENSG00000168310 | IRF2 | 1.07270759976747 | 2.39205637025769e-15 |
| ENSG00000117586 | TNFSF4 | 2.45787875154554 | 2.37699700584617e-15 |
| ENSG00000185885 | IFITM1 | 3.81942266976714 | 2.37502195403698e-15 |
| ENSG00000186470 | BTN3A2 | 1.93998114154565 | 2.36327086024061e-15 |
| ENSG00000196739 | COL27A1 | 1.33868994294433 | 2.23076410840349e-15 |
| ENSG00000104320 | NBN | 1.21034930348623 | 2.10895664501257e-15 |
| ENSG00000213886 | UBD | 7.68367061472836 | 1.83616707157697e-15 |
| ENSG00000116663 | FBXO6 | 2.99671315260107 | 1.40588176585438e-15 |

| | | | |
|---|---|---|---|
| ENSG00000155629 | PIK3AP1 | 5.19486531045649 | 1.30354074545694e-15 |
| ENSG00000122515 | ZMIZ2 | 1.07001694790357 | 1.20129597382369e-15 |
| ENSG00000085662 | AKR1B1 | 1.70641078744884 | 1.03646189650011e-15 |
| ENSG00000162772 | ATF3 | 3.21283385603815 | 9.90081914929569e-16 |
| ENSG00000130303 | BST2 | 5.66076385485507 | 9.4470286077385e-16 |
| ENSG00000161791 | FMNL3 | 1.20308594181872 | 9.3283227310742e-16 |
| ENSG00000104518 | GSDMD | 1.70726727573876 | 7.06765382657383e-16 |
| ENSG00000136436 | CALCOCO2 | 1.10152701552797 | 6.43943784826758e-16 |
| ENSG00000114270 | COL7A1 | 1.93161261713768 | 3.96771315590885e-16 |
| ENSG00000155363 | MOV10 | 1.3311626266151 | 3.93262734223342e-16 |
| ENSG00000131378 | RFTN1 | 1.05193813720617 | 3.43445246457531e-16 |
| ENSG00000140511 | HAPLN3 | 4.71439614965591 | 1.41118316420185e-16 |
| ENSG00000154451 | GBP5 | 9.48026120845911 | 8.19159836042755e-17 |
| ENSG00000138411 | HECW2 | 2.48508341324228 | 3.64456122264258e-17 |
| ENSG00000196954 | CASP4 | 1.04630521573991 | 3.44184695375514e-17 |
| ENSG00000106785 | TRIM14 | 1.79676143920438 | 2.96812395683679e-17 |
| ENSG00000041357 | PSMA4 | 1.34961664462438 | 2.93859575287364e-17 |
| ENSG00000096968 | JAK2 | 1.59188391980916 | 2.7866256816184e-17 |
| ENSG00000132109 | TRIM21 | 2.04971096286219 | 1.47846633519857e-17 |
| ENSG00000169429 | CXCL8 | 4.42780527247209 | 9.86048478350815e-18 |
| ENSG00000177989 | ODF3B | 4.19635973249736 | 8.44650252527789e-18 |
| ENSG00000131979 | GCH1 | 4.65045907188508 | 7.00133161120451e-18 |
| ENSG00000106366 | SERPINE1 | 1.82522893693592 | 4.84948143371885e-18 |
| ENSG00000004468 | CD38 | 7.78548990787374 | 4.32497991764467e-18 |
| ENSG00000134470 | IL15RA | 5.76611436037966 | 2.49516201903104e-18 |
| ENSG00000111859 | NEDD9 | 2.2289215550746 | 2.16307848324986e-18 |
| ENSG00000167996 | FTH1 | 1.32905670583257 | 2.15505265788208e-18 |
| ENSG00000108679 | LGALS3BP | 1.20798635797181 | 1.16875098526872e-18 |
| ENSG00000186088 | GSAP | 3.23968636029088 | 1.00766794450914e-18 |
| ENSG00000079385 | CEACAM1 | 8.46056838700829 | 8.54062454282667e-19 |
| ENSG00000183722 | LHFPL6 | 1.38170873956405 | 6.66222172363344e-19 |
| ENSG00000055332 | EIF2AK2 | 1.19375125679434 | 5.19574084350167e-19 |
| ENSG00000205220 | PSMB10 | 4.19670129496256 | 5.1273880630489e-19 |
| ENSG00000102524 | TNFSF13B | 3.76974237643683 | 4.96583426381239e-19 |
| ENSG00000056558 | TRAF1 | 4.01183101542365 | 4.92323597845198e-19 |
| ENSG00000157873 | TNFRSF14 | 3.97176414580032 | 2.61651940374175e-19 |
| ENSG00000125538 | IL1B | 2.38201418278132 | 2.21128564619922e-19 |
| ENSG00000166750 | SLFN5 | 1.29780097096986 | 1.909578494193e-19 |
| ENSG00000129667 | RHBDF2 | 2.19420216627218 | 1.14920347211113e-19 |
| ENSG00000130813 | SHFL | 1.68879340266814 | 8.01714752812414e-20 |
| ENSG00000239713 | APOBEC3G | 2.65968132583354 | 7.97112659409191e-20 |
| ENSG00000181381 | DDX60L | 2.17188587028225 | 5.99456424364974e-20 |
| ENSG00000041982 | TNC | 2.30343798581791 | 4.72575698229605e-20 |
| ENSG00000173391 | OLR1 | 8.35685965677046 | 2.94075991295605e-20 |
| ENSG00000145365 | TIFA | 2.1188973131453 | 2.37315067994406e-20 |
| ENSG00000100647 | SUSD6 | 1.93120688249085 | 1.54459620801828e-20 |

| | | | |
|---|---|---|---|
| ENSG00000184979 | USP18 | 3.87621790992504 | 1.04240620739015e-20 |
| ENSG00000196116 | TDRD7 | 1.71802199749789 | 5.81628519027628e-21 |
| ENSG00000109790 | KLHL5 | 1.10099962804017 | 5.53414044095343e-21 |
| ENSG00000154175 | ABI3BP | 2.92720385695161 | 4.40054409328974e-21 |
| ENSG00000104951 | IL4I1 | 8.54663807012643 | 1.29947671161656e-22 |
| ENSG00000204525 | HLA-C | 2.66012063242909 | 4.45861803196722e-23 |
| ENSG00000104635 | SLC39A14 | 1.49038047237895 | 3.67740136682372e-23 |
| ENSG00000136147 | PHF11 | 2.08521745543431 | 1.54818294250703e-23 |
| ENSG00000089127 | OAS1 | 10.2479365936309 | 8.33155352855343e-24 |
| ENSG00000170873 | MTSS1 | 2.79763054905567 | 7.51132082625029e-24 |
| ENSG00000155287 | SLC25A28 | 1.85168642903826 | 5.16089690176982e-24 |
| ENSG00000169871 | TRIM56 | 1.34040176382548 | 4.1792077225957e-24 |
| ENSG00000141574 | SECTM1 | 4.22856426013323 | 3.95499780540876e-24 |
| ENSG00000145779 | TNFAIP8 | 1.92823770262946 | 3.45123172029808e-24 |
| ENSG00000128641 | MYO1B | 1.1048948027327 | 1.9751054056828e-24 |
| ENSG00000135148 | TRAFD1 | 1.85716454310051 | 1.76120883304752e-24 |
| ENSG00000112149 | CD83 | 5.16501131990299 | 1.01998133566297e-24 |
| ENSG00000010030 | ETV7 | 8.27152203554633 | 5.12878156560055e-25 |
| ENSG00000187608 | ISG15 | 3.22153574195408 | 2.71909214884911e-25 |
| ENSG00000163874 | ZC3H12A | 2.40680740956907 | 2.66721727242909e-25 |
| ENSG00000151883 | PARP8 | 1.78163542343281 | 7.13567272780975e-26 |
| ENSG00000159110 | IFNAR2 | 2.03669402966012 | 2.37943349741837e-26 |
| ENSG00000013374 | NUB1 | 1.60872537470465 | 2.09893100477241e-26 |
| ENSG00000188313 | PLSCR1 | 2.2702778246311 | 5.32269417706003e-27 |
| ENSG00000173821 | RNF213 | 1.55152676469332 | 4.54774957818856e-27 |
| ENSG00000000971 | CFH | 1.97470217046361 | 2.92832445301866e-27 |
| ENSG00000134070 | IRAK2 | 2.86927505730572 | 2.18866616854532e-27 |
| ENSG00000131203 | IDO1 | 12.1983294435231 | 3.44091464756629e-28 |
| ENSG00000185745 | IFIT1 | 3.93037407860801 | 3.2831333447801e-28 |
| ENSG00000113583 | C5orf15 | 1.31217638194296 | 1.97972386289243e-28 |
| ENSG00000138642 | HERC6 | 3.97252207071333 | 1.27595830209767e-28 |
| ENSG00000185650 | ZFP36L1 | 1.23087793090158 | 6.94368747005189e-29 |
| ENSG00000263528 | IKBKE | 1.93237838567358 | 4.4604669284629e-29 |
| ENSG00000163735 | CXCL5 | 4.68405568627085 | 3.47358567347839e-29 |
| ENSG00000083799 | CYLD | 1.46155954808041 | 1.19110826634327e-29 |
| ENSG00000204267 | TAP2 | 3.12541688042484 | 4.38529601783333e-30 |
| ENSG00000113504 | SLC12A7 | 2.17816290131313 | 6.61945183852253e-31 |
| ENSG00000225492 | GBP1P1 | 7.81320288956448 | 6.42032069188467e-31 |
| ENSG00000166889 | PATL1 | 1.45966756865022 | 1.38275899227265e-31 |
| ENSG00000152778 | IFIT5 | 2.13488599654879 | 7.01927045901142e-32 |
| ENSG00000163131 | CTSS | 9.18577257817935 | 3.50765939665501e-32 |
| ENSG00000123609 | NMI | 2.58097487893455 | 3.16087004843448e-32 |
| ENSG00000130589 | HELZ2 | 2.56289422664009 | 2.70065661077673e-32 |
| ENSG00000003402 | CFLAR | 1.63579536154302 | 1.01364133680998e-32 |
| ENSG00000114127 | XRN1 | 1.39001843124391 | 6.13760961421964e-33 |
| ENSG00000138755 | CXCL9 | 13.2921418102202 | 1.85682277346774e-33 |

| | | | |
|---|---|---|---|
| ENSG00000170581 | STAT2 | 2.09917701409114 | 1.68583841278615e-33 |
| ENSG00000067066 | SP100 | 1.54345028393048 | 1.35327477451865e-33 |
| ENSG00000124226 | RNF114 | 1.32554093827002 | 9.04377699190007e-34 |
| ENSG00000197142 | ACSL5 | 10.0085187449485 | 5.99602044383844e-34 |
| ENSG00000256043 | CTSO | 2.10226747525224 | 2.81017700874576e-34 |
| ENSG00000109320 | NFKB1 | 1.98545103347661 | 2.21667429058999e-34 |
| ENSG00000168685 | IL7R | 3.93020112702902 | 3.23298977849887e-35 |
| ENSG00000180616 | SSTR2 | 10.1940646234242 | 2.14984184997327e-35 |
| ENSG00000117226 | GBP3 | 2.59869323376708 | 4.21871829332863e-36 |
| ENSG00000059378 | PARP12 | 3.16344416674437 | 1.58828260795694e-36 |
| ENSG00000163545 | NUAK2 | 3.17476950276546 | 1.48870645599373e-36 |
| ENSG00000162645 | GBP2 | 5.12178224306299 | 1.31426864829254e-36 |
| ENSG00000019582 | CD74 | 7.06181643167449 | 7.49593182299262e-37 |
| ENSG00000131323 | TRAF3 | 1.26800228023167 | 4.21913229562359e-38 |
| ENSG00000158714 | SLAMF8 | 10.3468235635817 | 1.89443976486202e-38 |
| ENSG00000126709 | IFI6 | 4.60180324277488 | 6.74284760295289e-39 |
| ENSG00000137496 | IL18BP | 3.70961736858384 | 2.0047920837363e-39 |
| ENSG00000271503 | CCL5 | 12.2401253591528 | 1.29730445122524e-39 |
| ENSG00000111335 | OAS2 | 9.56544927728301 | 5.13114253532536e-40 |
| ENSG00000121060 | TRIM25 | 2.02281014539828 | 2.61860189115969e-40 |
| ENSG00000163840 | DTX3L | 2.67932765221233 | 4.75029258164779e-41 |
| ENSG00000111331 | OAS3 | 4.70379604469726 | 4.09761159515042e-41 |
| ENSG00000206503 | HLA-A | 2.80168115783592 | 1.20018104690302e-41 |
| ENSG00000160710 | ADAR | 1.30670698928247 | 7.91786714022353e-42 |
| ENSG00000185215 | TNFAIP2 | 3.55971471162244 | 1.7520425548595e-42 |
| ENSG00000006118 | TMEM132A | 2.08801230856396 | 1.04436298393905e-42 |
| ENSG00000128335 | APOL2 | 2.79718049526518 | 2.81738632846888e-43 |
| ENSG00000116514 | RNF19B | 2.22769041859693 | 9.09307617149797e-44 |
| ENSG00000159403 | C1R | 3.31815471745847 | 2.38115155104595e-44 |
| ENSG00000169245 | CXCL10 | 12.4507666366978 | 2.34365297427136e-44 |
| ENSG00000136048 | DRAM1 | 2.83910775458683 | 2.06181557497099e-44 |
| ENSG00000169248 | CXCL11 | 12.6699701869505 | 2.03593825612241e-45 |
| ENSG00000137965 | IFI44 | 3.84126303796204 | 1.83573182377746e-45 |
| ENSG00000104856 | RELB | 3.46616112598545 | 2.34393326670285e-47 |
| ENSG00000104321 | TRPA1 | 3.35637235876021 | 3.00217807400477e-48 |
| ENSG00000023445 | BIRC3 | 7.38874300269848 | 2.39957399006317e-48 |
| ENSG00000137959 | IFI44L | 7.0566726203028 | 6.27982867182864e-49 |
| ENSG00000184371 | CSF1 | 2.48345085761036 | 2.02469507244434e-49 |
| ENSG00000160326 | SLC2A6 | 3.38014573404184 | 4.21538710195832e-50 |
| ENSG00000185880 | TRIM69 | 2.61096192449544 | 2.9242473920424e-50 |
| ENSG00000119922 | IFIT2 | 4.51065701846977 | 5.28115701232775e-51 |
| ENSG00000085117 | CD82 | 2.84697783913871 | 1.36245941970539e-51 |
| ENSG00000124201 | ZNFX1 | 2.22608679453458 | 1.24854430644958e-51 |
| ENSG00000101347 | SAMHD1 | 2.99499505347486 | 1.07217176220945e-51 |
| ENSG00000026950 | BTN3A1 | 2.25451323135286 | 2.79796672336031e-52 |
| ENSG00000137752 | CASP1 | 3.15617171515283 | 2.11028072259064e-52 |

| | | | |
|---|---|---|---|
| ENSG00000173918 | C1QTNF1 | 3.56888385120194 | 8.18089839910144e-53 |
| ENSG00000164342 | TLR3 | 3.7638137603315 | 7.51788651075023e-54 |
| ENSG00000111801 | BTN3A3 | 2.4510290789167 | 5.68336026834804e-55 |
| ENSG00000140853 | NLRC5 | 2.40971205865313 | 2.13756500545443e-55 |
| ENSG00000146457 | WTAP | 1.66396511027504 | 1.12057773811921e-56 |
| ENSG00000204264 | PSMB8 | 2.95897246661328 | 2.86162666716321e-60 |
| ENSG00000182179 | UBA7 | 2.79146909029159 | 4.43543294865818e-63 |
| ENSG00000117228 | GBP1 | 5.4849591278334 | 8.67661003387524e-64 |
| ENSG00000092010 | PSME1 | 2.32456571008615 | 2.45234257845555e-64 |
| ENSG00000164307 | ERAP1 | 2.39198919150681 | 3.30220047616486e-65 |
| ENSG00000118503 | TNFAIP3 | 4.08837560822987 | 1.57434813470824e-65 |
| ENSG00000110446 | SLC15A3 | 4.4245558340413 | 1.49632918705144e-65 |
| ENSG00000119917 | IFIT3 | 4.76585067325236 | 2.22461015981923e-66 |
| ENSG00000145901 | TNIP1 | 2.79712581165011 | 2.08542277185152e-67 |
| ENSG00000123240 | OPTN | 2.24541789808995 | 7.75162724578911e-69 |
| ENSG00000107201 | DDX58 | 3.26566827381875 | 3.92313240483679e-69 |
| ENSG00000100906 | NFKBIA | 3.13456287853344 | 2.93210391788634e-71 |
| ENSG00000140464 | PML | 2.16354453633286 | 1.22881743094025e-71 |
| ENSG00000050344 | NFE2L3 | 2.95463052152133 | 1.98841429857885e-73 |
| ENSG00000162692 | VCAM1 | 5.03332804326892 | 8.30486046398411e-75 |
| ENSG00000125347 | IRF1 | 5.05512845635621 | 6.87885248019072e-75 |
| ENSG00000132274 | TRIM22 | 2.68936285753241 | 5.75460027561119e-76 |
| ENSG00000138496 | PARP9 | 2.99069552748706 | 1.61400909563963e-77 |
| ENSG00000157601 | MX1 | 5.57083763195883 | 1.92874976343526e-78 |
| ENSG00000182326 | C1S | 4.1235454377124 | 7.77166505833095e-79 |
| ENSG00000068079 | IFI35 | 4.37730575003352 | 8.99245143075107e-80 |
| ENSG00000115267 | IFIH1 | 4.72791654709258 | 3.56460603219033e-81 |
| ENSG00000100342 | APOL1 | 5.26477424695378 | 1.78386444455822e-82 |
| ENSG00000133106 | EPSTI1 | 4.65526281679211 | 5.48342457253915e-83 |
| ENSG00000156587 | UBE2L6 | 2.86577078052323 | 1.62153555368822e-86 |
| ENSG00000132530 | XAF1 | 4.102424954281 | 3.29955550198887e-88 |
| ENSG00000162654 | GBP4 | 7.54104348108632 | 2.3080081599632e-88 |
| ENSG00000140105 | WARS1 | 3.35344663868919 | 4.19693620730509e-90 |
| ENSG00000077150 | NFKB2 | 2.9799789651188 | 2.00221518111186e-92 |
| ENSG00000240065 | PSMB9 | 5.5411448612068 | 1.2945094173056e-92 |
| ENSG00000221963 | APOL6 | 3.245179390496 | 1.63808455778275e-95 |
| ENSG00000090339 | ICAM1 | 7.45649573388841 | 6.95169938611548e-100 |
| ENSG00000100911 | PSME2 | 2.94085942094947 | 7.7576613378632e-101 |
| ENSG00000204592 | HLA-E | 2.54698204521068 | 1.22730484554262e-105 |
| ENSG00000108691 | CCL2 | 4.49669596154457 | 1.81477857837999e-107 |
| ENSG00000137628 | DDX60 | 3.83393209187308 | 1.25786987934563e-115 |
| ENSG00000112096 | SOD2 | 4.67315542586998 | 6.36270444588102e-123 |

| | | | |
|---|---|---|---|
| ENSG00000177409 | SAMD9L | 4.15856088363939 | 8.46806287780526e-127 |
| ENSG00000231925 | TAPBP | 2.42949524723615 | 1.03178025723561e-127 |
| ENSG00000166710 | B2M | 2.71130097266835 | 1.17164616417153e-132 |
| ENSG00000002549 | LAP3 | 3.53727103735013 | 2.85110428230042e-149 |
| ENSG00000025708 | TYMP | 4.61870923464223 | 2.25882707463123e-156 |
| ENSG00000173193 | PARP14 | 3.68831011027336 | 4.21244705297921e-188 |
| ENSG00000234745 | HLA-B | 5.12957393054837 | 3.16379099332301e-216 |
| ENSG00000168394 | TAP1 | 4.5538380222852 | 2.04449702082612e-231 |
| ENSG00000008517 | IL32 | 8.3130153873193 | 1.1608901310363e-231 |
| ENSG00000115415 | STAT1 | 4.38970246199904 | 3.78659217268625e-254 |
| ENSG00000004776 | HSPB6 | -1.29609188587191 | 5.74185010414917e-10 |
| ENSG00000006468 | ETV1 | -1.05733443121114 | 5.17685621827303e-10 |
| ENSG00000010278 | CD9 | -1.76713748196478 | 5.87302647994597e-11 |
| ENSG00000010438 | PRSS3 | -2.5386850182412 | 0.00017818689840293 |
| ENSG00000011201 | ANOS1 | -2.05933943127474 | 1.82726489866164e-05 |
| ENSG00000011347 | SYT7 | -2.64047565562867 | 1.8867478903155e-07 |
| ENSG00000013588 | GPRC5A | -2.40741113667698 | 1.37968351965679e-25 |
| ENSG00000019991 | HGF | -1.7893807875047 | 2.64889602411393e-10 |
| ENSG00000042493 | CAPG | -1.0928261131359 | 3.00032491282531e-08 |
| ENSG00000048342 | CC2D2A | -1.0194946278421 | 3.36900168362273e-05 |
| ENSG00000058668 | ATP2B4 | -1.27699794571778 | 1.18538185979832e-15 |
| ENSG00000059915 | PSD | -4.1814667832563 | 8.18442691320732e-05 |
| ENSG00000060656 | PTPRU | -1.10919809950933 | 3.2189677879694e-08 |
| ENSG00000067992 | PDK3 | -1.4316906914433 | 0.000105193907515794 |
| ENSG00000072195 | SPEG | -1.15262494115095 | 9.07827423203478e-06 |
| ENSG00000074181 | NOTCH3 | -1.64260781969597 | 7.89309912512986e-12 |
| ENSG00000076706 | MCAM | -1.15372106906888 | 0.000716889632013674 |
| ENSG00000085185 | BCORL1 | -1.06871973515412 | 2.79830938016938e-05 |
| ENSG00000091409 | ITGA6 | -1.65095890179867 | 7.86060154610608e-07 |
| ENSG00000091986 | CCDC80 | -1.03152438695977 | 6.10846455904164e-18 |
| ENSG00000092445 | TYRO3 | -1.18063967559179 | 1.87340128552746e-05 |
| ENSG00000095383 | TBC1D2 | -1.43431674943046 | 1.24877561439937e-11 |
| ENSG00000099204 | ABLIM1 | -2.3384775833805 | 2.39959607116407e-09 |
| ENSG00000099282 | TSPAN15 | -2.05173580023909 | 1.43789461926407e-05 |
| ENSG00000100321 | SYNGR1 | -1.66914348197994 | 0.000112400908766485 |
| ENSG00000100422 | CERK | -1.17007684165884 | 1.47649279151493e-08 |
| ENSG00000101187 | SLCO4A1 | -2.7356409239846 | 0.000506522111331839 |
| ENSG00000101265 | RASSF2 | -2.41102599134466 | 2.26920055852265e-05 |
| ENSG00000102271 | KLHL4 | -1.85121529602724 | 0.000149108524807892 |
| ENSG00000102468 | HTR2A | -4.10371540250057 | 0.000329100798825866 |

| | | | |
|---|---|---|---|
| ENSG00000102802 | MEDAG | -4.11443076215237 | 4.45419757022868e-08 |
| ENSG00000103485 | QPRT | -1.45767210660436 | 0.000189569752622686 |
| ENSG00000104081 | BMF | -5.80224836486112 | 0.000248139256461089 |
| ENSG00000104332 | SFRP1 | -1.60362384953858 | 2.08077125679311e-13 |
| ENSG00000104368 | PLAT | -2.42331034745487 | 4.57292752334556e-23 |
| ENSG00000104722 | NEFM | -3.77351116722761 | 3.31799045163523e-14 |
| ENSG00000105989 | WNT2 | -4.50284383713522 | 0.000423854783068011 |
| ENSG00000106123 | EPHB6 | -2.06870496500932 | 3.75497130656108e-07 |
| ENSG00000106976 | DNM1 | -1.16007592061685 | 6.41217875529436e-05 |
| ENSG00000108405 | P2RX1 | -3.69731757117002 | 0.00030699080178715 |
| ENSG00000108840 | HDAC5 | -1.00902799286653 | 0.000146092640950346 |
| ENSG00000108984 | MAP2K6 | -2.22569689807438 | 0.000465595659703554 |
| ENSG00000109099 | PMP22 | -1.26421851624948 | 7.60432521625996e-06 |
| ENSG00000109511 | ANXA10 | -1.59838737298488 | 4.77257754760392e-06 |
| ENSG00000109654 | TRIM2 | -1.04525297223505 | 9.34310736513525e-12 |
| ENSG00000110002 | VWA5A | -1.73231504021556 | 4.83952036868233e-05 |
| ENSG00000110400 | NECTIN1 | -1.06734561546812 | 1.85026719241319e-05 |
| ENSG00000110436 | SLC1A2 | -5.39571392075205 | 0.000257681129853472 |
| ENSG00000111057 | KRT18 | -1.41771366540011 | 8.50703177448805e-06 |
| ENSG00000113389 | NPR3 | -4.32317633857707 | 1.82203804713665e-05 |
| ENSG00000113594 | LIFR | -1.35245726524238 | 1.55524179857027e-16 |
| ENSG00000114115 | RBP1 | -1.1786415402599 | 0.000378592406454519 |
| ENSG00000115461 | IGFBP5 | -2.82839149776502 | 5.00093840217207e-07 |
| ENSG00000115525 | ST3GAL5 | -1.65819776549905 | 2.87343652333841e-19 |
| ENSG00000115648 | MLPH | -1.79367632491326 | 0.000110814308808374 |
| ENSG00000116133 | DHCR24 | -1.11121700277033 | 9.68155642309628e-06 |
| ENSG00000116285 | ERRFI1 | -1.06530005036834 | 5.63867843231309e-05 |
| ENSG00000116741 | RGS2 | -1.96107411082133 | 2.68962003052272e-09 |
| ENSG00000118785 | SPP1 | -2.84940079836584 | 2.32847185646834e-05 |
| ENSG00000119938 | PPP1R3C | -1.1265732985211 | 8.54988233399144e-07 |
| ENSG00000120693 | SMAD9 | -2.33111081663877 | 0.000592244008724887 |
| ENSG00000120885 | CLU | -1.03041516958625 | 9.89441382813047e-06 |
| ENSG00000121039 | RDH10 | -1.41270968843276 | 0.000272621794965456 |
| ENSG00000121904 | CSMD2 | -1.31232039456886 | 1.45846959777207e-06 |
| ENSG00000122778 | KIAA1549 | -1.13644280603156 | 1.03371459649169e-05 |
| ENSG00000123358 | NR4A1 | -2.23126016463383 | 1.83669052872085e-05 |
| ENSG00000124191 | TOX2 | -1.39482286752145 | 3.92430318565749e-05 |
| ENSG00000124920 | MYRF | -1.3238480783624 | 2.53219695798292e-06 |
| ENSG00000125378 | BMP4 | -1.91276489150991 | 0.00018894182054735 |
| ENSG00000125384 | PTGER2 | -1.87505703514883 | 2.80727082937512e-06 |
| ENSG00000125827 | TMX4 | -1.10237839646353 | 1.11129537477606e-11 |
| ENSG00000125848 | FLRT3 | -2.39660030819259 | 7.92270100174124e-07 |
| ENSG00000126016 | AMOT | -1.14403117652375 | 6.03953594749272e-05 |
| ENSG00000127418 | FGFRL1 | -1.37478374968091 | 9.82693532414861e-11 |
| ENSG00000128052 | KDR | -1.62375327258599 | 4.31546399837624e-05 |
| ENSG00000128564 | VGF | -5.39605826983748 | 0.000186449523859436 |

| | | | |
|---|---|---|---|
| ENSG00000130592 | LSP1 | -2.75567639757489 | 5.57445342433235e-06 |
| ENSG00000130600 | H19 | -4.00179436739813 | 2.71185168499707e-05 |
| ENSG00000131386 | GALNT15 | -2.8931301585319 | 0.000994758382017181 |
| ENSG00000132561 | MATN2 | -1.21337201688899 | 1.76860593231651e-07 |
| ENSG00000132563 | REEP2 | -1.60362359148023 | 0.000461278488820919 |
| ENSG00000134072 | CAMK1 | -1.13917880805943 | 5.23073175957468e-06 |
| ENSG00000134569 | LRP4 | -1.29006631707868 | 5.63584036507653e-06 |
| ENSG00000136928 | GABBR2 | -3.47045937080377 | 2.28672813812886e-06 |
| ENSG00000137501 | SYTL2 | -1.4859781292628 | 2.42325337560372e-05 |
| ENSG00000137642 | SORL1 | -1.62652602830283 | 0.000293080814065798 |
| ENSG00000138336 | TET1 | -1.25078226503612 | 2.45827751485965e-05 |
| ENSG00000138678 | GPAT3 | -2.53467860449104 | 1.62632945606738e-05 |
| ENSG00000139209 | SLC38A4 | -1.72567013930395 | 8.64539011258265e-06 |
| ENSG00000140015 | KCNH5 | -2.15938627302269 | 0.000614556709851106 |
| ENSG00000141469 | SLC14A1 | -4.52436745825709 | 3.17258124293311e-13 |
| ENSG00000143320 | CRABP2 | -3.09716042706822 | 1.68957690301427e-17 |
| ENSG00000143786 | CNIH3 | -1.9523147734776 | 0.000404176731377668 |
| ENSG00000143867 | OSR1 | -1.80778409308267 | 7.63997973179747e-05 |
| ENSG00000144136 | SLC20A1 | -1.14542309407219 | 6.40099198043804e-08 |
| ENSG00000144476 | ACKR3 | -1.16061074283743 | 0.000241472398937785 |
| ENSG00000144645 | OSBPL10 | -1.2154979333293 | 3.41098220255481e-07 |
| ENSG00000144730 | IL17RD | -1.57374255916069 | 0.000303587546796678 |
| ENSG00000145358 | DDIT4L | -2.54342004167968 | 1.0973102981764e-10 |
| ENSG00000145990 | GFOD1 | -1.53287617045211 | 0.000585614766234049 |
| ENSG00000146674 | IGFBP3 | -1.62848623279657 | 1.9596426665061e-07 |
| ENSG00000147251 | DOCK11 | -1.17439234199392 | 3.78025018248406e-11 |
| ENSG00000149090 | PAMR1 | -1.58202919696393 | 3.24123667281258e-08 |
| ENSG00000149150 | SLC43A1 | -1.59902865792536 | 9.7869049120083e-05 |
| ENSG00000149294 | NCAM1 | -1.91186037072793 | 7.841820148276e-10 |
| ENSG00000149451 | ADAM33 | -1.59830126065759 | 3.14386937582598e-07 |
| ENSG00000151136 | BTBD11 | -1.2399305137962 | 2.83003114826123e-06 |
| ENSG00000151150 | ANK3 | -1.1358347218725 | 8.95422906128502e-07 |
| ENSG00000151692 | RNF144A | -1.47522877215484 | 6.40326498354536e-10 |
| ENSG00000152127 | MGAT5 | -1.50877435922371 | 1.29532330992525e-12 |
| ENSG00000152580 | IGSF10 | -2.14797840524037 | 0.000418337906631958 |
| ENSG00000154122 | ANKH | -1.04639597138991 | 6.44608226518293e-08 |
| ENSG00000154217 | PITPNC1 | -1.58753141399515 | 3.85785925907433e-05 |
| ENSG00000154309 | DISP1 | -1.31586510280372 | 0.000329127091150822 |
| ENSG00000154639 | CXADR | -2.80453276976283 | 2.61736794151892e-07 |
| ENSG00000154734 | ADAMTS1 | -1.53387822065608 | 0.000119497337935859 |
| ENSG00000156103 | MMP16 | -1.42429954838836 | 4.71034430751114e-11 |
| ENSG00000156475 | PPP2R2B | -4.25507519704465 | 0.000484701157672848 |
| ENSG00000157168 | NRG1 | -1.26421547360211 | 6.87808675634686e-08 |
| ENSG00000157470 | FAM81A | -3.26510580324551 | 0.000176187913727747 |
| ENSG00000159167 | STC1 | -2.1879417963338 | 4.35734416215352e-05 |
| ENSG00000161714 | PLCD3 | -1.60954618631347 | 1.88472742378964e-11 |

| | | | |
|---|---|---|---|
| ENSG00000162076 | FLYWCH2 | -1.20519298059959 | 6.75427817105321e-06 |
| ENSG00000162545 | CAMK2N1 | -1.27579938212261 | 3.0885857944481e-05 |
| ENSG00000162595 | DIRAS3 | -1.88133722317435 | 1.12079810955927e-05 |
| ENSG00000163485 | ADORA1 | -1.99681994839578 | 0.000148676260382795 |
| ENSG00000164056 | SPRY1 | -3.15408971536469 | 0.000406627367798435 |
| ENSG00000164107 | HAND2 | -1.66890775007403 | 1.5125094705679e-10 |
| ENSG00000164251 | F2RL1 | -1.85321441541447 | 1.33082370466689e-05 |
| ENSG00000164850 | GPER1 | -2.36185451059296 | 0.000185572747292813 |
| ENSG00000165821 | SALL2 | -1.21141123240034 | 7.70293342811096e-07 |
| ENSG00000166106 | ADAMTS15 | -3.01206909966044 | 5.85573726864862e-07 |
| ENSG00000166396 | SERPINB7 | -2.04215829354011 | 3.4182971627225e-05 |
| ENSG00000166897 | ELFN2 | -2.33242640589103 | 3.02297501744819e-08 |
| ENSG00000168077 | SCARA3 | -1.29102864286445 | 2.65736389987463e-08 |
| ENSG00000168268 | NT5DC2 | -1.20583745028176 | 9.60319182102654e-09 |
| ENSG00000169439 | SDC2 | -1.18699538360129 | 4.95106372104796e-12 |
| ENSG00000170271 | FAXDC2 | -1.58945652757479 | 6.05995331156145e-05 |
| ENSG00000170500 | LONRF2 | -4.60126241155977 | 1.71913923972196e-08 |
| ENSG00000171033 | PKIA | -1.40107551778075 | 7.65790769212383e-06 |
| ENSG00000171208 | NETO2 | -1.13404389204951 | 1.03513007049729e-06 |
| ENSG00000171345 | KRT19 | -2.19096575979855 | 1.85329849102743e-19 |
| ENSG00000171408 | PDE7B | -1.09447422125702 | 4.68578470024403e-05 |
| ENSG00000171724 | VAT1L | -1.5905080913579 | 8.99284056446914e-07 |
| ENSG00000172020 | GAP43 | -1.89699322934688 | 3.35205821072963e-05 |
| ENSG00000172348 | RCAN2 | -5.76314946409263 | 8.76695504899359e-06 |
| ENSG00000172554 | SNTG2 | -4.27013136917344 | 0.000259104681597941 |
| ENSG00000173210 | ABLIM3 | -1.20893981230532 | 3.40538374029878e-08 |
| ENSG00000173530 | TNFRSF10D | -2.01067209531938 | 1.53504068841991e-17 |
| ENSG00000174600 | CMKLR1 | -3.00561559226357 | 0.000129384083809244 |
| ENSG00000174804 | FZD4 | -1.30018590983418 | 4.12070647331181e-08 |
| ENSG00000174807 | CD248 | -1.57015232199293 | 1.06580710837144e-09 |
| ENSG00000175866 | BAIAP2 | -1.11317345861615 | 0.000184197914427559 |
| ENSG00000176438 | SYNE3 | -1.18171938808066 | 1.32915411151006e-05 |
| ENSG00000176909 | MAMSTR | -1.80473572159389 | 0.000887944992831477 |
| ENSG00000178662 | CSRNP3 | -1.47934394921192 | 6.00690096894525e-05 |
| ENSG00000179388 | EGR3 | -2.13499011335024 | 0.000700458544112681 |
| ENSG00000181072 | CHRM2 | -3.02030078493172 | 0.0007620867058574 |
| ENSG00000182621 | PLCB1 | -1.70880002143837 | 1.05445073642152e-09 |
| ENSG00000184005 | ST6GALNAC3 | -1.48004902126042 | 5.69343237505762e-06 |
| ENSG00000185432 | METTL7A | -1.99507709913066 | 0.000498249805274523 |
| ENSG00000185551 | NR2F2 | -1.57348289571112 | 2.97294173574676e-25 |
| ENSG00000185585 | OLFML2A | -1.83283838113477 | 8.5473611934822e-13 |
| ENSG00000185630 | PBX1 | -1.29322023696493 | 9.53370718239379e-07 |
| ENSG00000185920 | PTCH1 | -1.54972657054395 | 3.86805524249612e-07 |
| ENSG00000187955 | COL14A1 | -1.75145176323401 | 0.000218675950228065 |
| ENSG00000188211 | NCR3LG1 | -1.0593396943948 | 5.31708223868143e-07 |
| ENSG00000188385 | JAKMIP3 | -2.87037166620088 | 0.00042236516134696 |

| | | | |
|---|---|---|---|
| ENSG00000188483 | IER5L | -1.20515159704749 | 0.000358273570842716 |
| ENSG00000189184 | PCDH18 | -1.11602480050623 | 1.98807380031776e-06 |
| ENSG00000196155 | PLEKHG4 | -1.95931751412963 | 5.30881131967673e-17 |
| ENSG00000196440 | ARMCX4 | -1.10384057574217 | 5.71990508562971e-07 |
| ENSG00000196460 | RFX8 | -1.19425394238886 | 1.59339301191678e-05 |
| ENSG00000196562 | SULF2 | -2.65918651092864 | 5.99905031296506e-07 |
| ENSG00000196814 | MVB12B | -1.11808013124313 | 0.000208803618088034 |
| ENSG00000197381 | ADARB1 | -1.00648678069284 | 7.20013805447836e-06 |
| ENSG00000197971 | MBP | -1.53689099903353 | 5.2783257120907e-06 |
| ENSG00000204131 | NHSL2 | -1.57406610055275 | 2.89715983059043e-09 |
| ENSG00000205213 | LGR4 | -1.50155439665083 | 3.01960045021924e-05 |
| ENSG00000205336 | ADGRG1 | -1.72546001174265 | 0.000453030564720647 |
| ENSG00000205978 | NYNRIN | -1.15233580634232 | 0.000332093711619168 |
| ENSG00000221890 | NPTXR | -1.6168615775978 | 2.13163066877306e-05 |
| ENSG00000221955 | SLC12A8 | -2.17832121362956 | 3.20715891979491e-09 |
| ENSG00000226950 | DANCR | -1.64430586820857 | 1.25028867207251e-10 |
| ENSG00000233384 | CNIH3-AS2 | -6.13969606401808 | 2.97101192646868e-09 |
| ENSG00000242265 | PEG10 | -1.45782415511102 | 3.05571006701568e-14 |
| ENSG00000243244 | STON1 | -1.44616332777011 | 0.000224844387521343 |
| ENSG00000243335 | KCTD7 | -1.11161357662066 | 0.000162828035882013 |
| ENSG00000245522 | LINC02709 | -5.23712394882415 | 0.000399642614231336 |
| ENSG00000268089 | GABRQ | -2.29001025487188 | 0.000105913662479048 |
| ENSG00000269190 | FBXO17 | -1.17569283837609 | 5.21430203143944e-06 |
| ENSG00000270885 | RASL10B | -2.48736346504715 | 0.000863697273296006 |
| ENSG00000271447 | MMP28 | -5.50886424889782 | 0.000945972658217407 |
| ENSG00000275342 | PRAG1 | -1.25827506673706 | 2.94557882061419e-08 |
| ENSG00000279192 | PWAR5 | -1.18139949200922 | 2.65361837733039e-05 |
