# Supplementary material for: The role of cytokine licensing in shaping the therapeutic potential of wharton’s jelly MSCs: metabolic shift towards immunomodulation at the expense of differentiation
Source: Stem Cell Res Ther. 2025 Apr 20;16:199. doi: 10.1186/s13287-025-04309-2 (PMC12010610; doi:10.1186/s13287-025-04309-2)
Supplement: Supplementary file 2 — Supplementary Material 2 [file 13287_2025_4309_MOESM2_ESM.pdf]

Additional file 2.

Table 1

The list of the genes related to MSC differentiation with log2 fold change in expression after cytokine priming, compared to unprimed cells, using a p-value threshold of 0.05, detected in RNA-seq results

Osteoblast differentiation GO:0001649

ENSEMBL	GENEID	log2FoldChange	pvalue
ENSG00000087085	ACHE	4.85749302266451	0.020262478254948
ENSG00000125845	BMP2	4.27998646180773	0.00409674498387892
ENSG00000041982	TNC	2.30343798581791	4.72575698229605e-20
ENSG00000162551	ALPL	2.27917166896795	0.0320013122407913
ENSG00000104415	CCN4	1.97129568132987	0.00531841199009953
ENSG00000138685	FGF2	1.36007699319376	1.19848461031997e-10
ENSG00000183160	TMEM119	1.31872307523768	0.0364702938543034
ENSG00000160710	ADAR	1.30670698928247	7.91786714022353e-42
ENSG00000170365	SMAD1	1.0759143876213	4.7740405865802e-06
ENSG00000081189	MEF2C	0.774513886695748	0.00818579624362319
ENSG00000142871	CCN1	0.750236052836474	0.0247772380399653
ENSG00000185386	MAPK11	0.707078895213713	0.0337201509666439
ENSG00000019549	SNAI2	0.699582487981592	1.52279508061079e-05
ENSG00000171223	JUNB	0.612244565154457	0.00471029283916264
ENSG00000166949	SMAD3	0.504414070077662	0.0342359710721317
ENSG00000133818	RRAS2	0.405512451689163	0.0393690044564627
ENSG00000075790	BCAP29	0.387850690534237	0.044531388217962
ENSG00000106571	GLI3	0.364845522277606	0.0364957497554673
ENSG00000011028	MRC2	0.214328580668008	0.0351393417613753
ENSG00000111875	ASF1A	-0.253147919173519	0.0235805616270689
ENSG00000147274	RBMX	-0.276379551689516	0.0276432400042659
ENSG00000113658	SMAD5	-0.374109793424235	0.000674060030920783
ENSG00000169710	FASN	-0.460883017061273	0.041279004849275
ENSG00000163041	H3-3A	-0.502652224171496	0.0104424352786242
ENSG00000108821	COL1A1	-0.564745486230836	0.0412327700336989
ENSG00000018408	WWTR1	-0.616510519405836	0.000948588323149331
ENSG00000121691	CAT	-0.630261335682497	0.000822930662834857
ENSG00000078804	TP53INP2	-0.716390230849522	0.00217824845917823
ENSG00000128602	SMO	-0.719407708658296	0.00589869230932885
ENSG00000152661	GJA1	-0.827192976453137	0.00149211803916635
ENSG00000138623	SEMA7A	-1.14830840897935	0.0374956853889182
ENSG00000205213	LGR4	-1.50155439665083	3.01960045021924e-05
ENSG00000104332	SFRP1	-1.60362384953858	2.08077125679311e-13
ENSG00000146674	IGFBP3	-1.62848623279657	1.9596426665061e-07
ENSG00000164107	HAND2	-1.66890775007403	1.5125094705679e-10
ENSG00000125378	BMP4	-1.91276489150991	0.00018894182054735
ENSG00000108984	MAP2K6	-2.22569689807438	0.000465595659703554

ENSG00000120693	SMAD9	-2.33111081663877	0.000592244008724887
ENSG00000172201	ID4	-2.5630983184631	0.00317274072853252
ENSG00000115461	IGFBP5	-2.82839149776502	5.00093840217207e-07
ENSG00000118785	SPP1	-2.84940079836584	2.32847185646834e-05

Fat cell differentiation GO:0045444

ENSEMBL	GENEID	log2FoldChange	pvalue
ENSG00000122877	EGR2	3.19159329173212	8.37304609387647e-13
ENSG00000204264	PSMB8	2.95897246661328	2.86162666716321e-60
ENSG00000164307	ERAP1	2.39198919150681	3.30220047616486e-65
ENSG00000185338	SOCS1	1.59397550164824	1.09598818915826e-05
ENSG00000169946	ZFPM2	1.15578291484027	0.00486912489640585
ENSG00000196878	LAMB3	0.954388243732133	0.00810895779162334
ENSG00000169136	ATF5	0.825821703462639	0.000683327570242982
ENSG00000019549	SNAI2	0.699582487981592	1.52279508061079e-05
ENSG00000172216	CEBPB	0.588672442740086	0.00324611239921008
ENSG00000173145	NOC3L	0.557345647095227	0.0272396459547281
ENSG00000140044	JDP2	0.495606956052022	0.0452834554733764
ENSG00000176845	METRNL	0.44276676313579	0.0443588464370925
ENSG00000171488	LRRC8C	0.367791857971638	0.0378599157407152
ENSG00000099968	BCL2L13	0.282781384863527	0.0163820886344737
ENSG00000091039	OSBPL8	-0.2419413912699	0.0162082427265708
ENSG00000163904	SENP2	-0.278392697550959	0.0301696835769399
ENSG00000148737	TCF7L2	-0.555823395725994	7.62122850472522e-06
ENSG00000179348	GATA2	-0.688083733714346	0.0284720006964565
ENSG00000112769	LAMA4	-1.12378100510084	0.0344554145405472
ENSG00000140876	NUDT7	-1.54037785343446	0.0396570657406303
ENSG00000182621	PLCB1	-1.70880002143837	1.05445073642152e-09
ENSG00000116741	RGS2	-1.96107411082133	2.68962003052272e-09
ENSG00000123358	NR4A1	-2.23126016463383	1.83669052872085e-05
ENSG00000221818	EBF2	-2.33934096664262	0.00512548824278946
ENSG00000174483	BBS1	-2.48513193206393	0.042771093014257
ENSG00000172201	ID4	-2.5630983184631	0.00317274072853252
ENSG00000132170	PPARG	-2.87663640511516	0.0295381459614236
ENSG00000154153	RETREG1	-3.11573633376234	0.00301105515516901

Chondrocyte differentiation (GO:0002062)

ENSEMBL	GENEID	log2FoldChange	pvalue
ENSG00000125845	BMP2	4.27998646180773	0.00409674498387892
ENSG00000110693	SOX6	1.79072340704559	1.76670280195522e-06
ENSG00000104635	SLC39A14	1.49038047237895	3.67740136682372e-23
ENSG00000196739	COL27A1	1.33868994294433	2.23076410840349e-15
ENSG00000137573	SULF1	1.07875005195979	0.00474984242399998
ENSG00000081189	MEF2C	0.774513886695748	0.00818579624362319

ENSG00000163513	TGFBR2	0.384843201397753	0.00502100121082317
ENSG00000106571	GLI3	0.364845522277606	0.0364957497554673
ENSG00000159216	RUNX1	0.25489429786351	0.0272771949476002
ENSG00000151348	EXT2	0.239526224457944	0.0156973373222898
ENSG00000182158	CREB3L2	-0.246314239344765	0.0183657598253923
ENSG00000149257	SERPINH1	-0.301353686470117	0.00919943989847761
ENSG00000100644	HIF1A	-0.375977193248252	0.0349973152943527
ENSG00000005073	HOXA11	-0.433550901129277	0.0244738691212015
ENSG00000105329	TGFB1	-0.464197350939149	0.00211091433392248
ENSG00000168056	LTBP3	-0.510074763215151	0.00220216643449691
ENSG00000120708	TGFBI	-0.539820170291062	0.043910085708359
ENSG00000172819	RARG	-0.775693732758197	0.00166836217896398
ENSG00000125398	SOX9	-0.877304858766358	0.00166227480564924
ENSG00000143867	OSR1	-1.80778409308267	7.63997973179747e-05
ENSG00000125378	BMP4	-1.91276489150991	0.00018894182054735
ENSG00000196562	SULF2	-2.65918651092864	5.99905031296506e-07
ENSG00000068078	FGFR3	-4.9495556692535	0.0319592486121285

Myoblast differentiation (GO:0045445)

ENSEMBL	GENEID	log2FoldChange	pvalue
ENSG00000168334	XIRP1	6.88999699069697	0.02117122897421
ENSG00000141052	MYOCD	1.54621110008814	8.42442441756778e-07
ENSG00000152601	MBNL1	-0.214943352047535	0.0290547606510064
ENSG00000148400	NOTCH1	-0.368070493446849	0.0181464271628603
ENSG00000178385	PLEKHM3	-0.445082121105524	0.0364919222084984
ENSG00000121068	TBX2	-0.456879531585258	0.0282401281156591
ENSG00000148737	TCF7L2	-0.555823395725994	7.62122850472522e-06
ENSG00000115884	SDC1	-0.686926732356757	0.00044446384231633
ENSG00000069011	PITX1	-0.864804647628793	0.00113542647821533
ENSG00000145794	MEGF10	-1.00466120819655	0.0425626558733762
ENSG00000157168	NRG1	-1.26421547360211	6.87808675634686e-08

Skeletal muscle cell differentiation (GO:0035914)

ENSEMBL	GENEID	log2FoldChange	pvalue
ENSG00000162772	ATF3	3.21283385603815	9.90081914929569e-16
ENSG00000122877	EGR2	3.19159329173212	8.37304609387647e-13
ENSG00000102554	KLF5	1.89572730638731	2.17060183367843e-08
ENSG00000185022	MAFF	0.830780250729529	0.00777800147002513
ENSG00000108179	PPIF	0.531471544986585	0.00172552190493227
ENSG00000127124	HIVEP3	0.491030863225936	0.0256773046737468
ENSG00000170802	FOXN2	0.490476842360495	0.0065595234167449
ENSG00000161904	LEMD2	0.482406024140338	0.00317631318144399
ENSG00000102119	EMD	-0.322618504451655	0.0390271152212813
ENSG00000148400	NOTCH1	-0.368070493446849	0.0181464271628603

ENSG00000164442	CITED2	-0.418688498475379	0.0326117535645614
ENSG00000120738	EGR1	-0.483488261311292	0.0312514550594062
ENSG00000064309	CDON	-0.847084946030669	0.0400341254467591
ENSG00000145794	MEGF10	-1.00466120819655	0.0425626558733762
ENSG00000123358	NR4A1	-2.23126016463383	1.83669052872085e-05

Smooth muscle cell differentiation (GO:0051145)

ENSEMBL	GENEID	log2FoldChange	pvalue
ENSG00000141052	MYOCD	1.54621110008814	8.42442441756778e-07
ENSG00000148926	ADM	1.16335462901883	0.0180886181536864
ENSG00000081189	MEF2C	0.774513886695748	0.00818579624362319
ENSG00000163069	SGCB	0.691891731707533	1.86014124628519e-09
ENSG00000172638	EFEMP2	0.431688291692517	0.000303867568306156
ENSG00000112531	QKI	0.256428759162378	0.0115499369441929
ENSG00000148400	NOTCH1	-0.368070493446849	0.0181464271628603
ENSG00000121068	TBX2	-0.456879531585258	0.0282401281156591
ENSG00000135111	TBX3	-0.7199817073094	7.76484160892341e-09
ENSG00000125398	SOX9	-0.877304858766358	0.00166227480564924
ENSG00000125378	BMP4	-1.91276489150991	0.00018894182054735
ENSG00000114315	HES1	-2.13201086719944	0.00110357460714982

Neuron differentiation (GO:0030182)

ENSEMBL	GENEID	log2FoldChange	pvalue
ENSG00000006210	CX3CL1	6.67219124827696	5.99252516676366e-06
ENSG00000125730	C3	6.56607126153943	2.94518189268112e-10
ENSG00000058866	DGKG	5.64762648124016	3.91137852007211e-11
ENSG00000162692	VCAM1	5.03332804326892	8.30486046398411e-75
ENSG00000065675	PRKCQ	4.76815634007519	0.0239396855523737
ENSG00000112096	SOD2	4.67315542586998	6.36270444588102e-123
ENSG00000136244	IL6	4.61689708836973	1.15160816040538e-11
ENSG00000125845	BMP2	4.27998646180773	0.00409674498387892
ENSG00000065320	NTN1	3.63373522454441	0.000118874086667775
ENSG00000122877	EGR2	3.19159329173212	8.37304609387647e-13
ENSG00000134198	TSPAN2	3.0342609795147	3.26776080184334e-06
ENSG00000187764	SEMA4D	2.42390578916788	3.06199439090422e-07
ENSG00000041982	TNC	2.30343798581791	4.72575698229605e-20
ENSG00000168280	KIF5C	2.22533957399358	0.0123956485067943
ENSG00000189056	RELN	2.11006911869201	0.00299297136897048
ENSG00000107485	GATA3	1.9703690958591	0.0270166408722776
ENSG00000143494	VASH2	1.69112528768826	0.010355926442566
ENSG00000096968	JAK2	1.59188391980916	2.7866256816184e-17
ENSG00000196358	NTNG2	1.54004113425707	2.83553812630185e-05
ENSG00000153993	SEMA3D	1.5243007729297	2.32045634482412e-07
ENSG00000138685	FGF2	1.36007699319376	1.19848461031997e-10

ENSG00000149571	KIRREL3	1.31701979978555	0.00128442137525482
ENSG00000163251	FZD5	1.27368872913647	0.0463548188788397
ENSG00000254087	LYN	1.2500257418608	1.03136949066006e-06
ENSG00000213949	ITGA1	1.2442292902673	0.000845922945581372
ENSG00000122641	INHBA	1.23933681172777	0.0164050105780646
ENSG00000164877	MICALL2	1.23012389303661	2.92895228209296e-05
ENSG00000135905	DOCK10	1.19967111782259	1.49372869202019e-10
ENSG00000148926	ADM	1.16335462901883	0.0180886181536864
ENSG00000145147	SLIT2	1.1417857260431	5.93283002898002e-15
ENSG00000079215	SLC1A3	1.03277767644374	0.00842594762081238
ENSG00000143013	LMO4	0.973858968162056	9.58752564725731e-08
ENSG00000101680	LAMA1	0.955108540322846	0.00274803525734153
ENSG00000152894	PTPRK	0.926052383865454	5.59283741660229e-14
ENSG00000108797	CNTNAP1	0.888124867448612	8.00980715999405e-07
ENSG00000118257	NRP2	0.883495900446085	2.09448242188623e-10
ENSG00000155760	FZD7	0.878638531492899	0.000316687444905028
ENSG00000113657	DPYSL3	0.85064529089849	7.17050978073738e-09
ENSG00000110911	SLC11A2	0.809927408927758	5.10313427399266e-05
ENSG00000152332	UHMK1	0.799866402285498	7.68060081802572e-06
ENSG00000000457	SCYL3	0.785602986694983	0.000947886725599985
ENSG00000058272	PPP1R12A	0.776151901968769	1.24274802947087e-05
ENSG00000081189	MEF2C	0.774513886695748	0.00818579624362319
ENSG00000128656	CHN1	0.770779995987216	0.000548837959927098
ENSG00000148154	UGCG	0.75842742097236	9.54709023260711e-05
ENSG00000163697	APBB2	0.727795124027559	2.15131257399098e-05
ENSG00000169855	ROBO1	0.71521966596851	1.58436316056455e-06
ENSG00000167100	SAMD14	0.7103376949978	0.0281784454530379
ENSG00000149177	PTPRJ	0.699984304082007	3.41269782904491e-07
ENSG00000151694	ADAM17	0.674928866446654	8.85215521061728e-05
ENSG00000110047	EHD1	0.67044697560956	0.000576988977728734
ENSG00000105894	PTN	0.654812427307933	0.0100168597123318
ENSG00000047579	DTNBP1	0.653244033067978	0.00495773119325605
ENSG00000158470	B4GALT5	0.627998020612395	9.68353668607276e-06
ENSG00000033178	UBA6	0.614193026174961	4.77627100287337e-07
ENSG00000105402	NAPA	0.605847568673366	0.000272592446685916
ENSG00000182704	TSKU	0.596898689562991	4.17369929627494e-05
ENSG00000165801	ARHGEF40	0.595854980607116	0.0040833061357861
ENSG00000136848	DAB2IP	0.59412694210137	0.000433378301008914
ENSG00000172216	CEBPB	0.588672442740086	0.00324611239921008
ENSG00000005810	MYCBP2	0.539328732473083	8.95459842391293e-09
ENSG00000068366	ACSL4	0.521396779186478	1.81109651524034e-05
ENSG00000173786	CNP	0.510994239582007	0.00405367842328272
ENSG00000162614	NEXN	0.500177098399416	0.000209737564846313
ENSG00000104884	ERCC2	0.500038948558779	0.00538620596546309
ENSG00000146648	EGFR	0.49674914521567	0.000179593209844366
ENSG00000160145	KALRN	0.493832364122264	0.0395254066260341

ENSG00000188157	AGRN	0.491149561506439	0.00763876942473615
ENSG00000196159	FAT4	0.483739393760975	0.0189065606620693
ENSG00000038382	TRIO	0.463536984228375	4.73315956622276e-06
ENSG00000143545	RAB13	0.44186114202218	0.00361307745773308
ENSG00000067141	NEO1	0.437779919610076	0.0294728779850269
ENSG00000087245	MMP2	0.433595795937506	0.00185890054211504
ENSG00000084733	RAB10	0.430161184775812	4.94343198575014e-06
ENSG00000010810	FYN	0.416953021191989	0.0301682906639927
ENSG00000077254	USP33	0.389557950316083	0.00025937575434822
ENSG00000118263	KLF7	0.374999898387029	0.028287854331835
ENSG00000168610	STAT3	0.36599332800483	0.00667497036789326
ENSG00000106571	GLI3	0.364845522277606	0.0364957497554673
ENSG00000133104	SPART	0.361298892863982	0.00252664106348408
ENSG00000136754	ABI1	0.348739279377586	0.00213038454386986
ENSG00000130479	MAP1S	0.34842188216361	0.0497147240301693
ENSG00000115317	HTRA2	0.347811310855197	0.0481096115043108
ENSG00000072518	MARK2	0.341217163863948	0.0320683776499774
ENSG00000073921	PICALM	0.315606394502079	0.0181261005958397
ENSG00000166483	WEE1	0.30990972812562	0.0188116513027845
ENSG00000103769	RAB11A	0.303947117934452	0.000541484269218773
ENSG00000142192	APP	0.297894296511739	0.00197021701637766
ENSG00000159216	RUNX1	0.25489429786351	0.0272771949476002
ENSG00000124486	USP9X	0.222588040518837	0.0176721479466528
ENSG00000170315	UBB	0.218107098225395	0.0366310635267175
ENSG00000179295	PTPN11	-0.192869381686945	0.0383736388701187
ENSG00000004487	KDM1A	-0.229122550899558	0.0284889139289441
ENSG00000163785	RYK	-0.233901407238352	0.0212075359233236
ENSG00000196591	HDAC2	-0.236871502596888	0.0211493793066763
ENSG00000140575	IQGAP1	-0.2426201338124	0.000255914885845086
ENSG00000164924	YWHAZ	-0.244766649922172	0.00587411969716484
ENSG00000035403	VCL	-0.245636304120401	0.0261272293032126
ENSG00000141736	ERBB2	-0.268777841737841	0.0425764759875335
ENSG00000154277	UCHL1	-0.276315728348655	0.0127547936006252
ENSG00000099250	NRP1	-0.278961804546603	0.00828443938192726
ENSG00000087053	MTMR2	-0.296078419554499	0.0158405975217378
ENSG00000082701	GSK3B	-0.296157870492772	0.0330465166428242
ENSG00000138814	PPP3CA	-0.299960276107933	0.00130181181645766
ENSG00000131238	PPT1	-0.322608854865144	0.0034739522080379
ENSG00000127445	PIN1	-0.328862293321525	0.0332342402357583
ENSG00000135541	AHI1	-0.34138276412835	0.0032152616231319
ENSG00000101752	MIB1	-0.345157169620529	0.000491932340038638
ENSG00000187240	DYNC2H1	-0.357508108516085	0.0232640811347956
ENSG00000148400	NOTCH1	-0.368070493446849	0.0181464271628603
ENSG00000130669	PAK4	-0.37143331167244	0.0222759078515293
ENSG00000100644	HIF1A	-0.375977193248252	0.0349973152943527
ENSG00000140443	IGF1R	-0.379545141255605	0.0441589266198748

ENSG00000163558	PRKCI	-0.389892707402675	7.72014707786318e-05
ENSG00000151247	EIF4E	-0.413007187356143	0.0266984812073126
ENSG00000129116	PALLD	-0.422221939373378	0.0494917254903923
ENSG00000112367	FIG4	-0.433754724818792	0.0258926349265508
ENSG00000107404	DVL1	-0.453662354832642	0.021496305039683
ENSG00000176101	SSNA1	-0.459055056474499	0.0313590398328507
ENSG00000110367	DDX6	-0.460751747887414	4.71185331654964e-06
ENSG00000070018	LRP6	-0.477671886297669	0.0035469175081825
ENSG00000071054	MAP4K4	-0.478639759249063	0.000243238515895383
ENSG00000130827	PLXNA3	-0.492404008899072	0.00296305404539124
ENSG00000162599	NFIA	-0.496287443010545	0.00447661731730598
ENSG00000133318	RTN3	-0.496516175639093	1.53889425705712e-05
ENSG00000152767	FARP1	-0.505376479367328	1.62435137791687e-08
ENSG00000197956	S100A6	-0.506435056376227	0.00269280928256346
ENSG00000091136	LAMB1	-0.519447871424557	8.92907457685241e-08
ENSG00000177732	SOX12	-0.538977234008865	0.0128543537435844
ENSG00000116132	PRRX1	-0.550698046252777	0.0022018558665896
ENSG00000115232	ITGA4	-0.556462422859994	0.00895408618823001
ENSG00000180818	HOXC10	-0.578071823138488	0.0132116771589915
ENSG00000167081	PBX3	-0.593145656932037	0.00101875384298619
ENSG00000112902	SEMA5A	-0.597291417564493	0.010290223190212
ENSG00000142949	PTPRF	-0.607938417254003	5.97809971052346e-07
ENSG00000106799	TGFBR1	-0.6519672536127	9.38414201699677e-05
ENSG00000163832	ELP6	-0.655700095538694	0.00948662301510163
ENSG00000166963	MAP1A	-0.658350189373116	6.21235473936265e-05
ENSG00000251493	FOXD1	-0.670814507002391	0.0039018165550082
ENSG00000136720	HS6ST1	-0.680374426188271	0.000806912294370678
ENSG00000179348	GATA2	-0.688083733714346	0.0284720006964565
ENSG00000135414	GDF11	-0.711319040930962	0.00586468155296469
ENSG00000144711	IQSEC1	-0.725318759439691	1.41753996753736e-05
ENSG00000169750	RAC3	-0.766443856386877	0.00297645738954827
ENSG00000147852	VLDLR	-0.768222112481649	0.00203159712687567
ENSG00000166313	APBB1	-0.782353164012385	0.00562102198166511
ENSG00000105290	APLP1	-0.80691257988602	0.015108294438653
ENSG00000133216	EPHB2	-0.838639117480839	0.0104768936597173
ENSG00000156299	TIAM1	-0.838956654988362	0.000963776339807028
ENSG00000064309	CDON	-0.847084946030669	0.0400341254467591
ENSG00000149256	TENM4	-0.854649645640061	0.000451015726912983
ENSG00000125398	SOX9	-0.877304858766358	0.00166227480564924
ENSG00000174684	B4GAT1	-0.918214741194831	3.79759499555568e-05
ENSG00000179542	SLITRK4	-0.985240465460577	0.0351654498652081
ENSG00000007237	GAS7	-1.00096154919236	0.00393990006430477
ENSG00000197381	ADARB1	-1.00648678069284	7.20013805447836e-06
ENSG00000006468	ETV1	-1.05733443121114	5.17685621827303e-10
ENSG00000167702	KIFC2	-1.05894147852121	0.00630367930686408
ENSG00000110400	NECTIN1	-1.06734561546812	1.85026719241319e-05

ENSG00000170989	S1PR1	-1.09608352272116	0.0111392652110244
ENSG00000060656	PTPRU	-1.10919809950933	3.2189677879694e-08
ENSG00000151150	ANK3	-1.1358347218725	8.95422906128502e-07
ENSG00000138623	SEMA7A	-1.14830840897935	0.0374956853889182
ENSG00000169439	SDC2	-1.18699538360129	4.95106372104796e-12
ENSG00000129757	CDKN1C	-1.20925727743442	0.00928472773018215
ENSG00000132561	MATN2	-1.21337201688899	1.76860593231651e-07
ENSG00000179242	CDH4	-1.2329445696295	0.00131862666282728
ENSG00000184347	SLIT3	-1.23536236687707	0.0087604489774236
ENSG00000154654	NCAM2	-1.2398242366763	0.00133053738968494
ENSG00000138336	TET1	-1.25078226503612	2.45827751485965e-05
ENSG00000008056	SYN1	-1.28228703972152	0.0202687237759615
ENSG00000134569	LRP4	-1.29006631707868	5.63584036507653e-06
ENSG00000185630	PBX1	-1.29322023696493	9.53370718239379e-07
ENSG00000184349	EFNA5	-1.38299269857664	0.00950431127665607
ENSG00000182580	EPHB3	-1.49739088128274	0.00130232144011529
ENSG00000120756	PLS1	-1.5373254973653	0.0319546636653355
ENSG00000185920	PTCH1	-1.54972657054395	3.86805524249612e-07
ENSG00000139304	PTPRQ	-1.56137639857904	0.0113681243579874
ENSG00000012171	SEMA3B	-1.59602736017213	0.028999797399657
ENSG00000104332	SFRP1	-1.60362384953858	2.08077125679311e-13
ENSG00000164107	HAND2	-1.66890775007403	1.5125094705679e-10
ENSG00000107562	CXCL12	-1.7341504507673	0.00683396599300556
ENSG00000172020	GAP43	-1.89699322934688	3.35205821072963e-05
ENSG00000149294	NCAM1	-1.91186037072793	7.841820148276e-10
ENSG00000125378	BMP4	-1.91276489150991	0.00018894182054735
ENSG00000196155	PLEKHG4	-1.95931751412963	5.30881131967673e-17
ENSG00000011201	ANOS1	-2.05933943127474	1.82726489866164e-05
ENSG00000106123	EPHB6	-2.06870496500932	3.75497130656108e-07
ENSG00000188064	WNT7B	-2.07415238272628	0.00339603744672605
ENSG00000114315	HES1	-2.13201086719944	0.00110357460714982
ENSG00000117707	PROX1	-2.15784781667062	0.0145556199124075
ENSG00000168477	TNXB	-2.20365805495592	0.0122427540091949
ENSG00000137872	SEMA6D	-2.25335797707502	0.0443923692868532
ENSG00000171798	KNDC1	-2.27630073315342	0.0278140881006652
ENSG00000174483	BBS1	-2.48513193206393	0.042771093014257
ENSG00000125968	ID1	-2.51248791612862	0.00669329967154298
ENSG00000172201	ID4	-2.5630983184631	0.00317274072853252
ENSG00000104435	STMN2	-2.77433079692217	0.00112485274730947
ENSG00000059915	PSD	-4.1814667832563	8.18442691320732e-05
ENSG00000105989	WNT2	-4.50284383713522	0.000423854783068011
