# Supplementary material for: The role of cytokine licensing in shaping the therapeutic potential of wharton’s jelly MSCs: metabolic shift towards immunomodulation at the expense of differentiation
Source: Stem Cell Res Ther. 2025 Apr 20;16:199. doi: 10.1186/s13287-025-04309-2 (PMC12010610; doi:10.1186/s13287-025-04309-2)
Supplement: Supplementary file 3 — Supplementary Material 3 [file 13287_2025_4309_MOESM3_ESM.pdf]

Additional file 3.

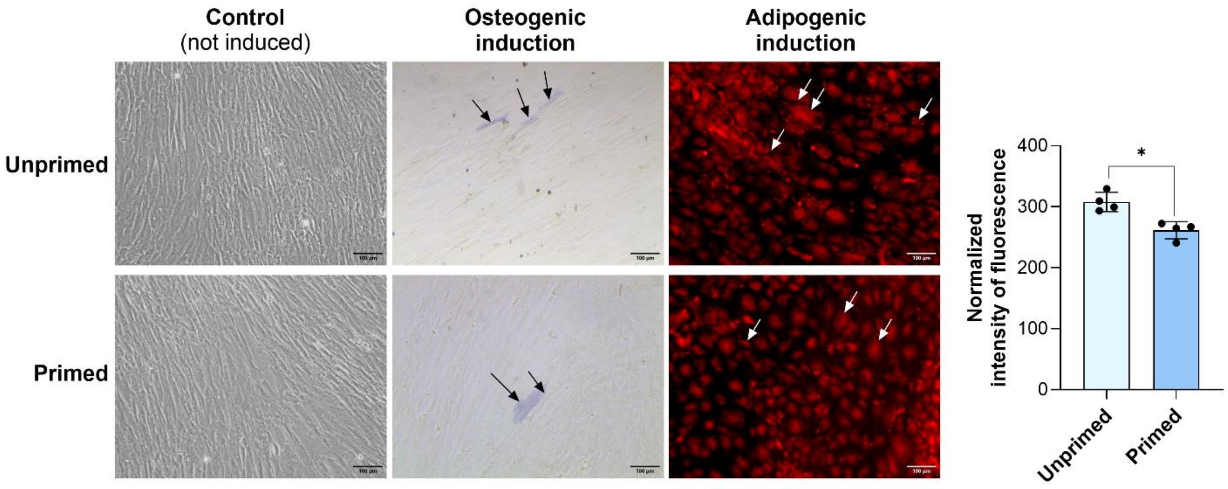

Figure 1S. Morphological properties and indicators of osteogenic (alkaline phosphatase staining, black arrows indicate the positive cells) and adipogenic (Nile red staining – white arrows indicate the lipid droplets) induction of unprimed (upper row) and primed (lower row) WJ-MSCs. The graph shows the quantitative analysis of adipogenic differentiation assessed by the intensity of Nile red staining, normalized to the number of cells (100,000 cells).

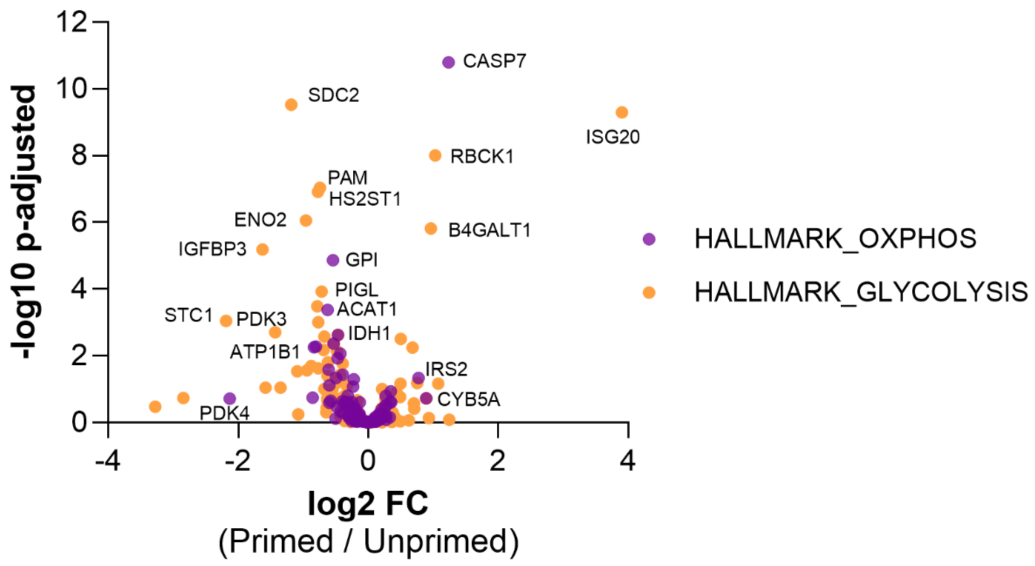

Figure 2S. The volcano plot comparing gene sets related to HALLMARK_OXPHOS (oxidative phosphorylation) and HALLMARK_GLYCOLYSIS (glycolysis) in primed and unprimed WJ-MSCs. The x-axis represents the log2 fold change (FC), while the y-axis shows the -log10 p-adjusted values. Each dot indicates a differentially expressed gene after priming.

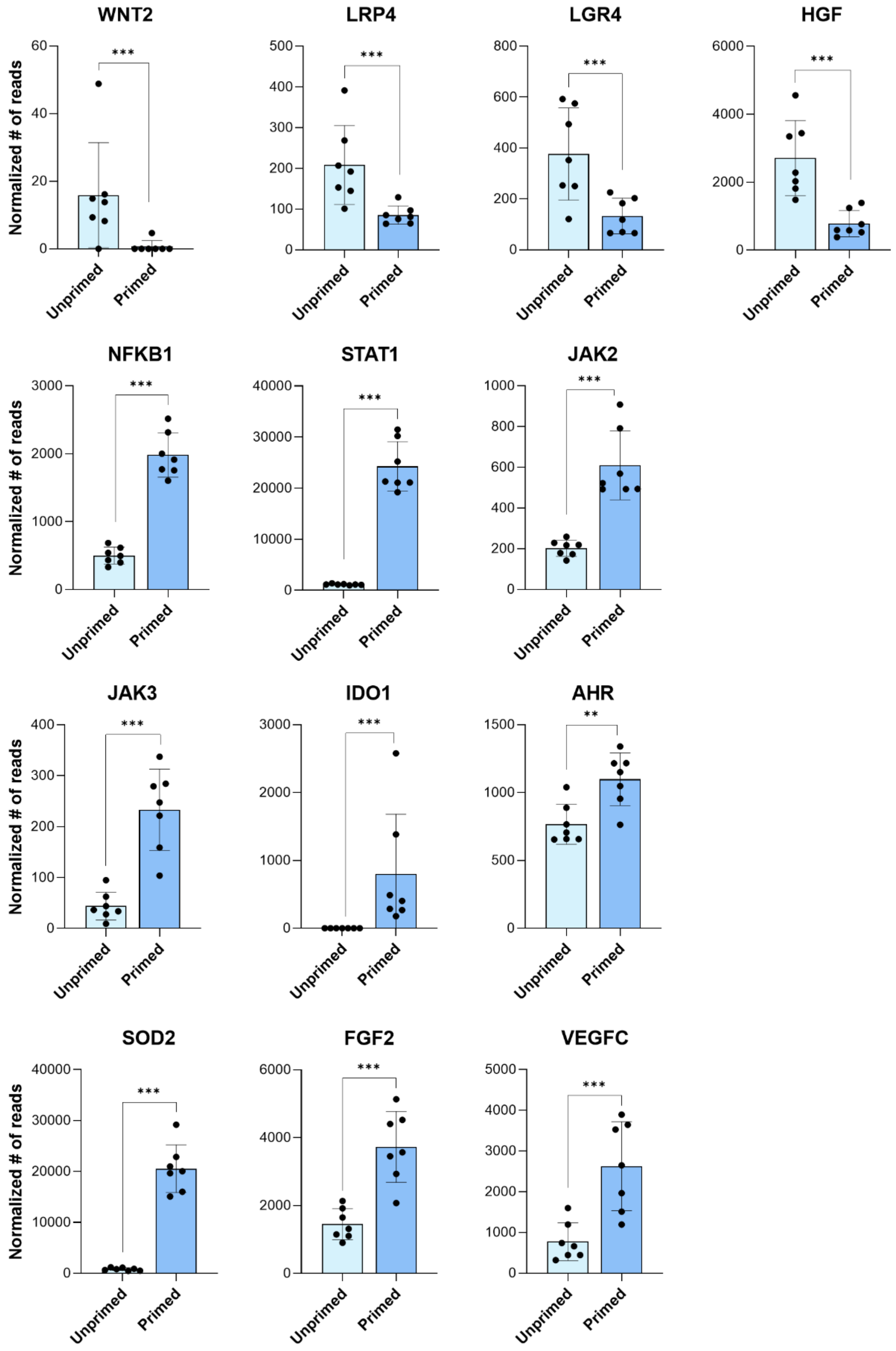

Figure 3S. Changes in the expression of genes of interest after short-term cytokine licensing. Note: ** - p<0.01; *** - p<0.001.

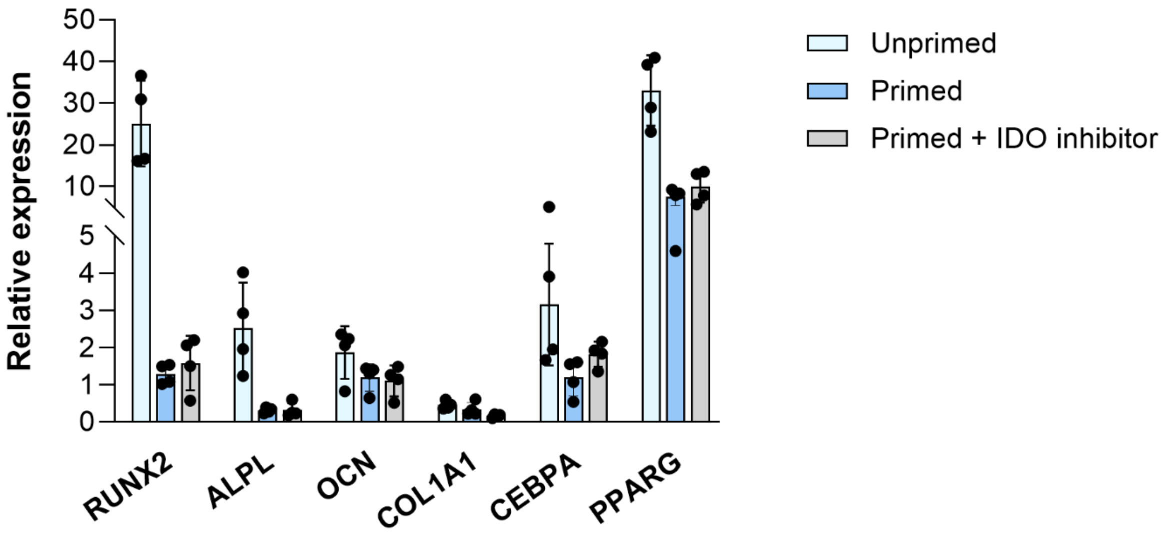

Figure 4S. The expression of osteogenic and adipogenic genes of unprimed and cytokine licensed WJ-MSCs (N=4 in duplicates) with and without IDO inhibition by 1-methyl-DL-tryptophan (1-MT, 1 mM) following the 7 days of the differentiation induction. Data is presented as Mean ± SD relative expression of induced cells to control cells (not subjected to differentiation induction) Ns – non-significant; * - $p<0.05$; ** - $p<0.01$.

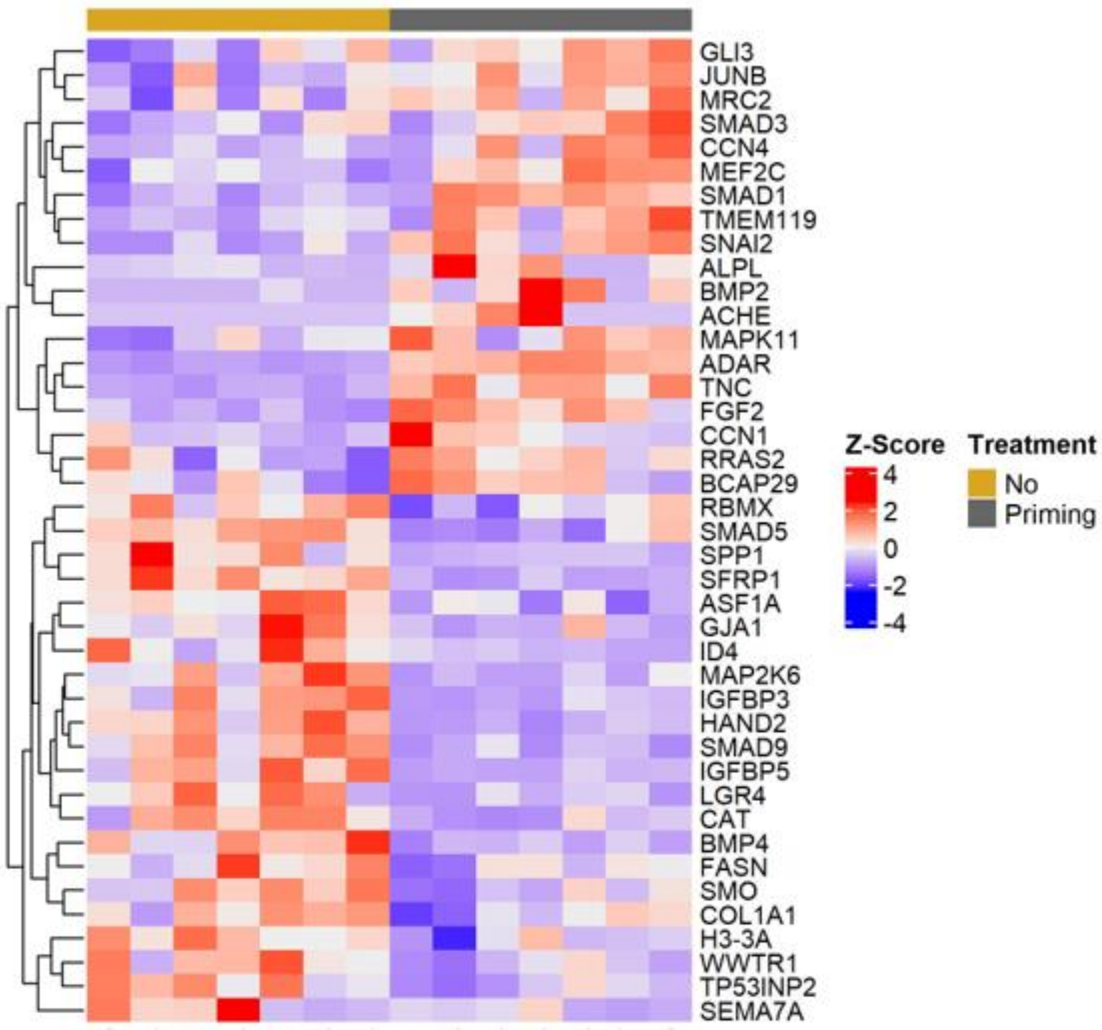

Figure 5S. Heatmap of changes occurred in the expression of genes related to osteogenic differentiation. The genes with a log2 fold change in expression after cytokine priming, compared to unprimed cells, were identified according to GO:0001649 (osteoblast differentiation), using a p-value threshold of 0.05.

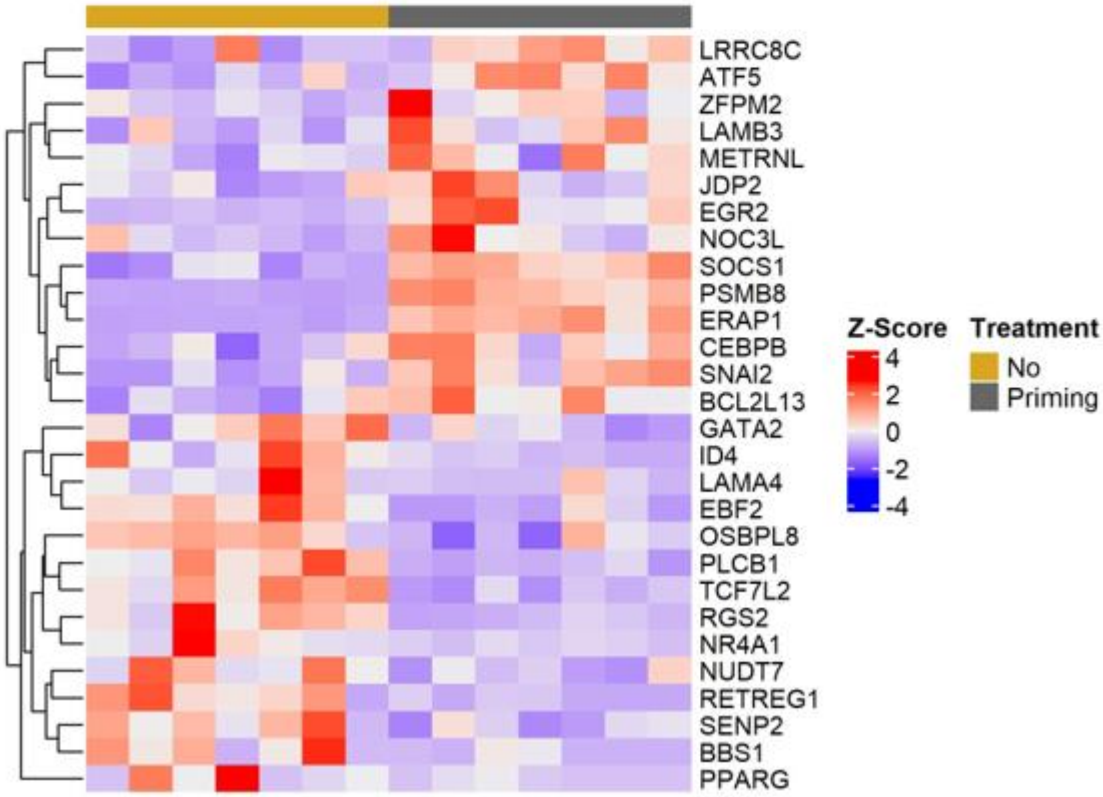

Figure 6S. Heatmap of changes occurred in the expression of genes related to adipogenic differentiation. The genes with a log2 fold change in expression after cytokine priming, compared to unprimed cells, were identified according to GO:0045444 (fat cell differentiation), using a p-value threshold of 0.05.
